# Comparing the efficacy of chlorhexidine and povidone-iodine for surgical site disinfection: a systematic review and meta-analysis from randomized controlled trials

**DOI:** 10.1080/07853890.2026.2634531

**Published:** 2026-02-26

**Authors:** Lei Wang, Shenghao Xu, Qibo Xu, Jianlin Xiao

**Affiliations:** ^a^ Department of Orthopedics, China-Japan Union Hospital of Jilin University, Changchun, Jilin, China; ^b^ Department of Orthopedics, The Second Hospital of Jilin University, Changchun, Jilin, China

**Keywords:** Povidone-iodine, chlorhexidine, surgical site infection, meta-analysis

## Abstract

**Background:**

Randomized controlled trials report conflicting evidence on the efficacy of different skin disinfectants for preventing surgical site infection (SSI).

**Methods:**

We systematically searched PubMed, Web of Science, Cochrane Library, and Embase for RCTs published up to February 2025 comparing preoperative skin disinfection with povidone-iodine (PVI) versus chlorhexidine (CH). Primary outcomes were overall, superficial, deep, and organ/space SSI rates. Secondary outcomes included hospital stay, readmission, and reoperation.

**Results:**

CH was superior to PVI in preventing overall SSI (26 studies, *n* = 29,356; RR: 0.89; 95% confidence interval [CI]: 0.80 to 0.99). The overall SSI incidence rate in the CH group was 7.1% (1,045/14,677), compared with 7.8% (1,152/14,679) in the PVI group, equating to an 11% reduction in relative risk and a 0.7% reduction in absolute risk. The number needed to treat to prevent one SSI was 143. CH demonstrated superiority over PVI in preventing superficial SSI (13 studies, *n* = 16,867; RR: 0.77; 95% CI: 0.64 to 0.92), but not for deep SSI (11 studies, *n* = 15,842; RR: 1.00; 95% CI: 0.77 to 1.29) or organ SSI (9 studies, *n* = 9,471; RR: 1.17; 95% CI: 0.89 to 1.53). No significant differences were found in hospital stay, readmission, or reoperation rates between the two groups.

**Conclusion:**

CH demonstrates statistical superiority over PVI in preventing overall and superficial SSI, though the absolute clinical benefit is modest. No significant differences were observed for deep or organ/space SSI, nor for secondary outcomes including hospital length of stay, readmission, or reoperation rates.

## Introduction

Surgical site infection is defined as an infection occurring at or near the surgical incision site following surgery [1]. Most cases result from inadequate preoperative skin disinfection of the patient, allowing residual pathogens at the wound site to proliferate extensively and cause postoperative infection [2]. The severity of surgical site infection (SSI) varies depending on the type of surgery, ranging from simple purulent wound discharge to life-threatening conditions [3]. Additionally, postoperative complications resulting from SSI prolong hospital stays [1], leading to extended antibiotic use. In some cases, patients with poor prognoses may require readmission, thereby increasing unnecessary healthcare expenditures [4]. In the most developed country, the United States, annual healthcare expenditures for SSI reach as high as $10 billion. Despite such substantial investment, the outcomes remain far from satisfactory. SSI still accounts for 20% of all hospital-acquired infections in the United States [5]. Moreover, patients’ hospital stays are prolonged due to SSIs or require readmission because of infection [5]. In the most underdeveloped regions of Africa, SSIs are the most common postoperative complication, with infection-related deaths accounting for as high as 9.7% of all cases [6].

The polyvinylpyrrolidone component in povidone-iodine (PVI) exhibits affinity for cell membranes, enabling it to introduce the active ingredient iodine to the surface of bacterial cell membranes. It then attacks the bacterial cell membrane and cytoplasmic membrane [7]. PVI disrupts the integrity of bacterial cell membranes and can also penetrate cells to interfere with normal physiological metabolic processes, rapidly killing bacteria within seconds. It demonstrates effective results against both Gram-positive and Gram-negative bacteria. Compared to conventional iodine solutions, PVI remains at the wound site, slowly and continuously releasing its active ingredient, iodine, to prevent the occurrence of postoperative SSI [8]. An epidemiological study has also shown that methicillin-resistant Staphylococcus aureus (MRSA) exhibits a tendency to lower its susceptibility, which can lead to high mortality and infection rates among patients [9]. However, few epidemiological studies have been performed to show pathogen resistance to PVI. It can kill pathogens such as MRSA within 20–30 s and leaves fewer bacteria at the wound site [10].

Chlorhexidine (CH), with its positive charge, binds to bacterial cell membranes, which changes the permeability of the membranes and causes them to lose their function. It also inhibits the activity of enzymes involved in bacterial energy metabolism, thus preventing normal bacterial reproduction. Gram-positive bacteria are more sensitive to CH because they have no outer membrane barrier, compared to Gram-negative bacteria, making it easier for CH to enter the bacterial interior [11]. Past meta-analysis has demonstrated that residual CH alcohol solution can lower the incidence of postoperative SSIs by staying on the skin surface, slowly releasing active ingredients, and continuing to inhibit bacterial growth and reproduction for several hours [12]. Previous studies indicate that smvA, initially described as a major facilitator superfamily (MFS) transporter encoded on the chromosome, confers bacterial resistance by reducing drug concentrations within the cell [13]. A recent study has shown that the increased resistance of Klebsiella pneumoniae to chlorhexidine correlates with the upregulation of the efflux pump gene smvA, which originates from its adjacent reverse transcription gene smvR [14]. The rising incidence of Klebsiella pneumoniae infections may lead to increased mortality rates, higher treatment costs, and a heavier social and economic burden [15].

The above arguments demonstrate that the bactericidal efficacy of chlorhexidine is compromised by the upregulation of resistance genes such as smvA, while epidemiological evidence regarding the potential for systematic resistance to povidone-iodine remains insufficient. Additionally, multiple randomized controlled trials indicate that both chlorhexidine and povidone-iodine demonstrate distinct clinical advantages in reducing SSI [16–41]. However, existing meta-analyses have primarily concentrated on comparing the direct efficacy of chlorhexidine and povidone-iodine in preventing SSI, while a comprehensive evaluation of their overall clinical benefits in real-world practice remains limited. Therefore, this study aims to perform a meta-analysis to systematically evaluate their combined clinical utility, following a comparative assessment of their disinfection efficacy.

## Methods

### Search strategy and eligibility criteria

We used the keywords ‘antisepsis,’ ‘chlorhexidine,’ ‘povidone-iodine,’ and ‘surgery’ to conduct a search in PubMed, Web of Science, Cochrane Library, and Embase database till February 2025. A search strategy that combined the keywords was implemented in the Supplementary method. For the studies that met the initial inclusion criteria, we adopted a manual retrieval method to include them. We also screened references to the identified articles. We searched clinical trial registries and contacted investigators *via* email to obtain unpublished data. Then, based on the PICOS principle, the studies that were re-included were successively excluded, and finally the studies that would be included in the systematic review and meta-analysis were determined in Table S1.

This systematic review and meta-analysis were conducted according to the Preferred Reporting Items for Systematic Reviews and Meta-Analyses (PRISMA) guidelines [42] and A Measurement Tool to Assess Systematic Reviews 2 (AMSTAR2) guidelines [43]. The meta-analysis was registered on PROSPERO prospectively (Registry number: CRD420251136491). The pre-specified analysis was conducted based on the registration date of 29 August 2025.

### Data extraction

To ensure accuracy, dual independent data extraction was performed by two authors (LW and QBX) using Microsoft Excel 2019. The investigators worked in isolation without communication regarding the extraction process until both had finalized their datasets. Two researchers (LW and QBX) initially screened the retrieved literature by reviewing the titles and abstracts using the reference management software EndNote 21 to identify eligible studies. Any discrepancies during the full-text screening process were resolved by a third author (JLX). Two authors (LW and SHX) independently conducted data extraction from the eligible studies, with inclusion determined based on the PICOS criteria. Demographic and clinical characteristics of the participants were extracted. The participant characteristics included demographic variables such as age and sex ratio. Data on primary and secondary outcomes were extracted from the eligible publications, including tabulated results and graphical data.

### Assessment of methodological quality

To assess the risk of bias in the included studies, two reviewers (WL and QBX) independently applied the Cochrane Risk of Bias Assessment Tool, Version 2 (RoB2) [44]. Assessment focused on five domains: the randomization process (D1), deviations from intended interventions (D2), missing outcome data (D3), outcome measurement (D4), and selection of the reported result (D5). Each domain, and subsequently the overall study, was judged as having a “Low risk,” “Some concerns,” or “High risk” of bias. The overall risk of bias was classified according to a pre-defined hierarchy: a study was rated “High risk” if any domain was judged at high risk, or if both the randomization (D1) and outcome measurement (D4) domains were rated as having some concerns. A rating of ‘Some concerns’ was assigned when any domain was rated with some concerns, provided the criteria for “High risk” were not met. “Low risk” required all domains to be rated as low risk. Disagreements in ratings were resolved through discussion, with arbitration by a third reviewer (JLX) if consensus could not be reached.

In this systematic review, we assessed the quality of evidence for both primary and secondary outcomes using the Grading of Recommendations Assessment, Development and Evaluation (GRADE) approach.

### Statistics analysis

Dichotomous outcomes were expressed as risk ratios (RR) with 95% confidence intervals (CI), while continuous outcomes were recorded as mean and standard deviation (SD). At the same time, the continuous outcomes were analyzed using standardized mean differences (SMDs).

The pooled effect estimates were calculated using a Mantel-Haenszel random-effects model [45] and presented with traditional forest plots. Statistical heterogeneity between studies was evaluated with the I^2^ statistic, where I^2^ > 50% indicated substantial heterogeneity, and I^2^ ≤ 50% suggested moderate or low heterogeneity. A *P*-value of < 0.05 with a 95% confidence interval was considered statistically significant.

The primary outcomes were the incidence rates of overall SSI, superficial SSI, deep SSI and organ SSI. The secondary outcomes were length of hospital stay, incidence of readmission, and incidence of reoperation. Our definition of SSI is based on the standards proposed by the U.S. Centers for Disease Control and Prevention (CDC), and according to the CDC standards, SSI is classified into three subtypes: superficial SSI, deep SSI and organ SSI [46]. For primary and secondary outcomes, we conducted subgroup analyses and sensitivity analyses to identify potential sources of heterogeneity. The underlying publication bias was assessed using the Egger’s test [47]. All statistical analyses were performed using Review Manager software, version 5.4, and Stata software, version 12.0.

## Results

**Figure 1.**
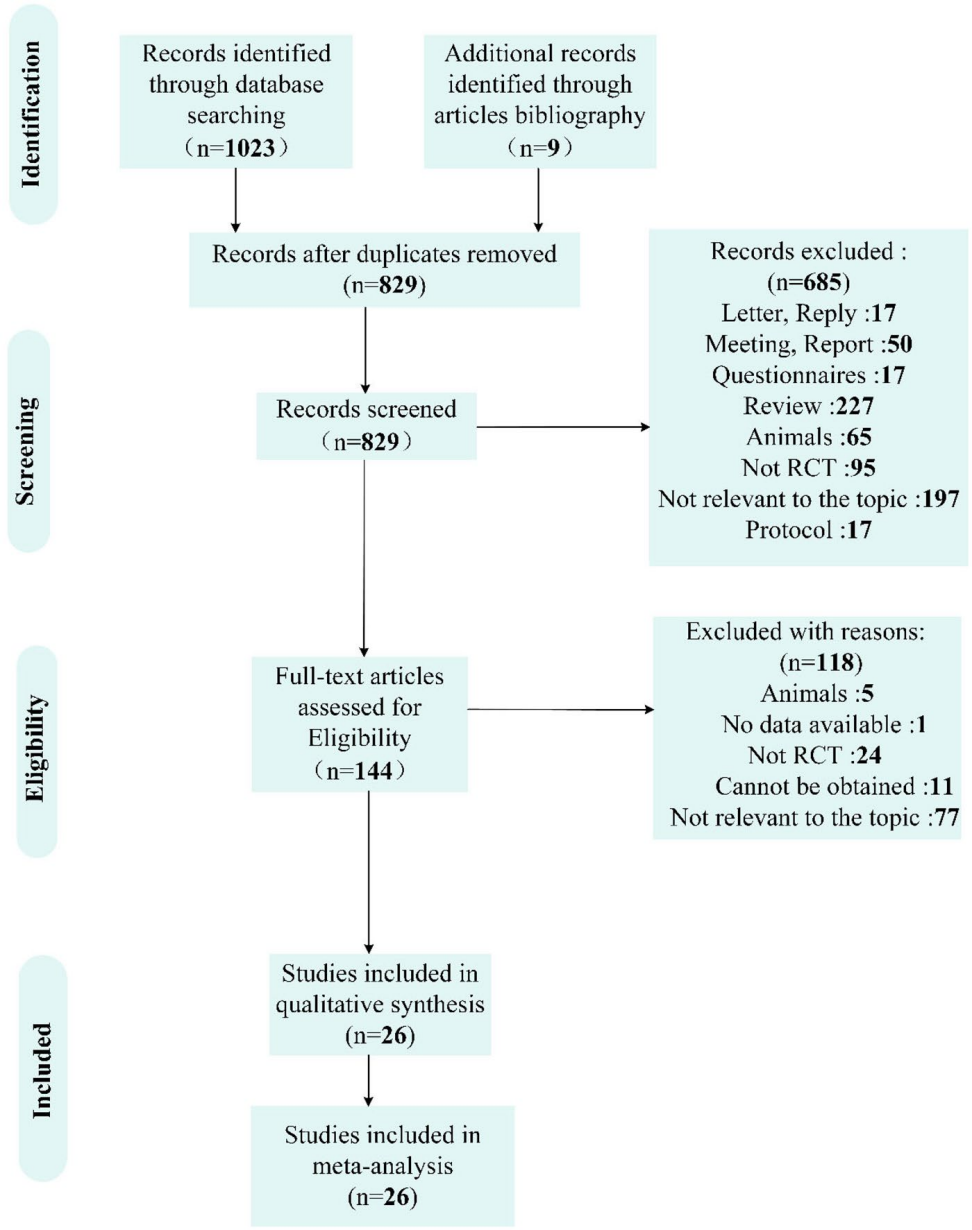
The selection process of the included randomized clinical trials.

Our systematic search identified 1,023 records from databases, with 9 additional articles retrieved through reference tracking. After removing 203 duplicates and 685 publications that failed to meet inclusion criteria based on title/abstract screening. Following comprehensive database retrieval and manual screening procedures, 144 eligible studies were selected for thorough full-text examination. After rigorous evaluation, 26 eligible studies qualified for inclusion in the final meta-analysis [16–41] (Figure 1).

### Methodological quality of included studies

We assessed the quality of the 26 included randomized controlled trials (RCTs) using RoB2. The results of the risk of bias analyses are shown in Figure S1. Using the RoB2 tool for assessment, 25 of the 26 included studies were rated as “low risk” [17–41], with only one study rated as “some concerns” [16].

### Incidence of overall SSI

**Figure 2.**
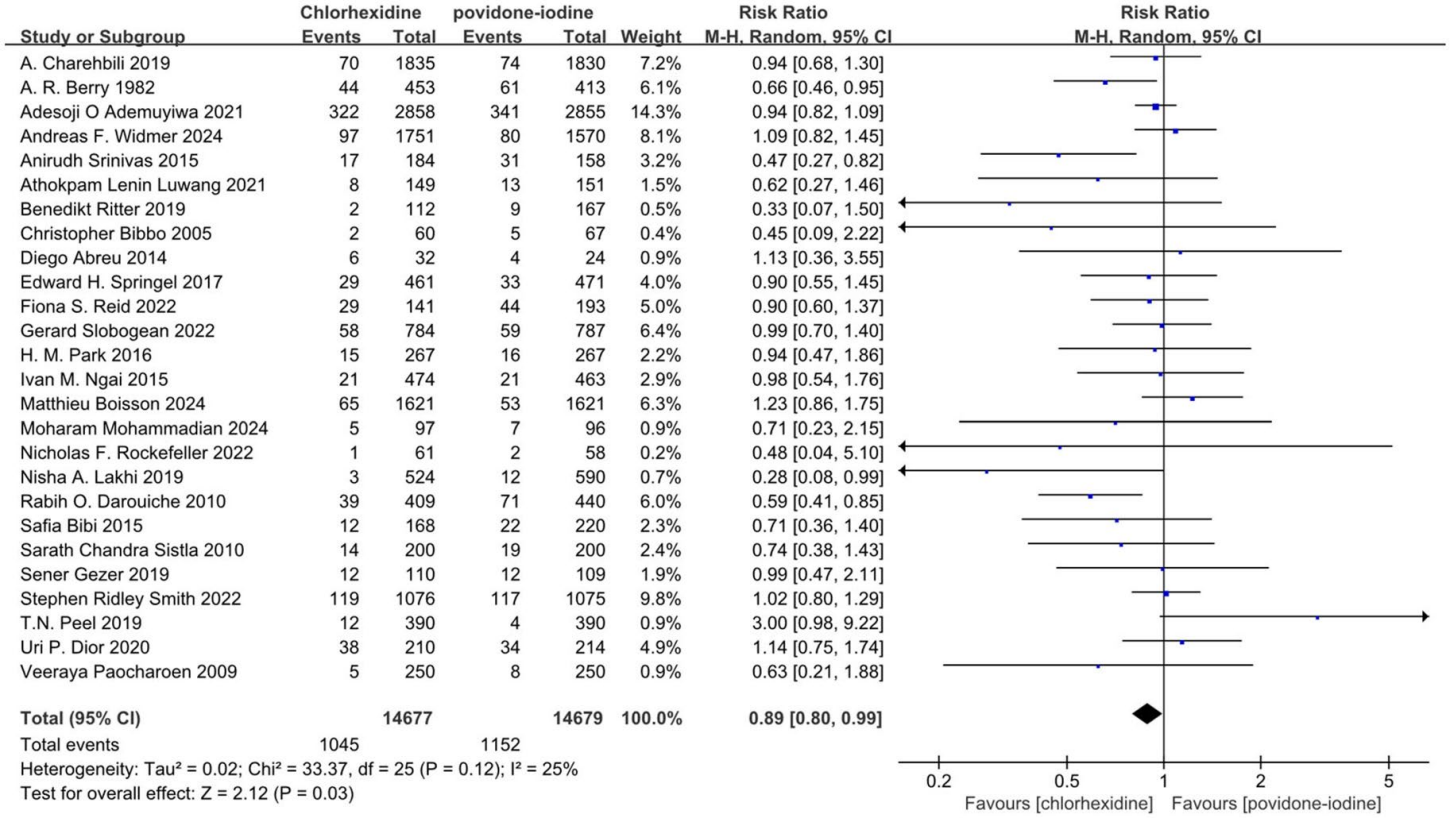
Forest plot of the incidence of overall SSI included in the meta-analysis. SSI, surgical site infection; M-H, Mantel-Haenszel; CI, confidence interval.

All twenty-six studies utilized overall SSI as the primary endpoint [16–41]. The collective findings suggest that CH was superior to PVI in preventing overall SSI in Table S2 and Figure 2 (RR: 0.89; 95% CI: 0.80 to 0.99; I^2^ = 25%; *n* = 29,356; *P* = 0.03). The symmetry of the funnel plot and the results of Egger’s test did not reveal any significant evidence of publication bias in Table S8 and Figure S2.

### Incidence of superficial SSI, deep SSI and organ SSI

**Figure 3.**
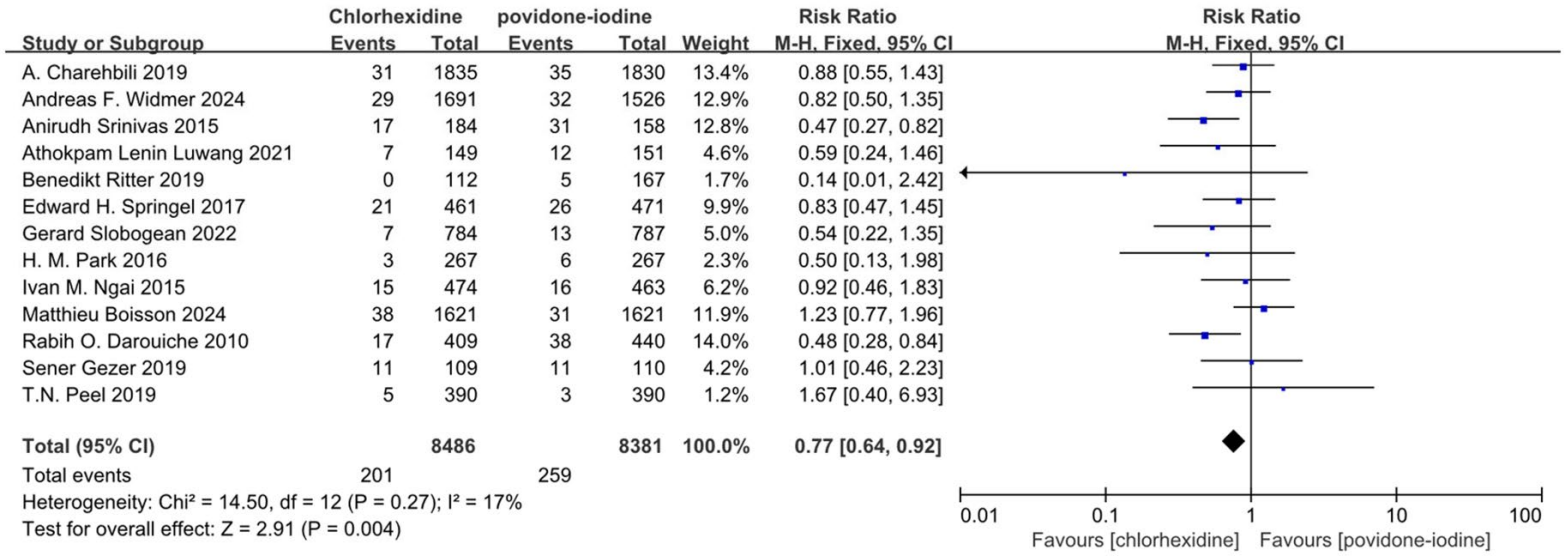
Forest plot of the incidence of superficial SSI included in the meta-analysis.

**Figure 4.**
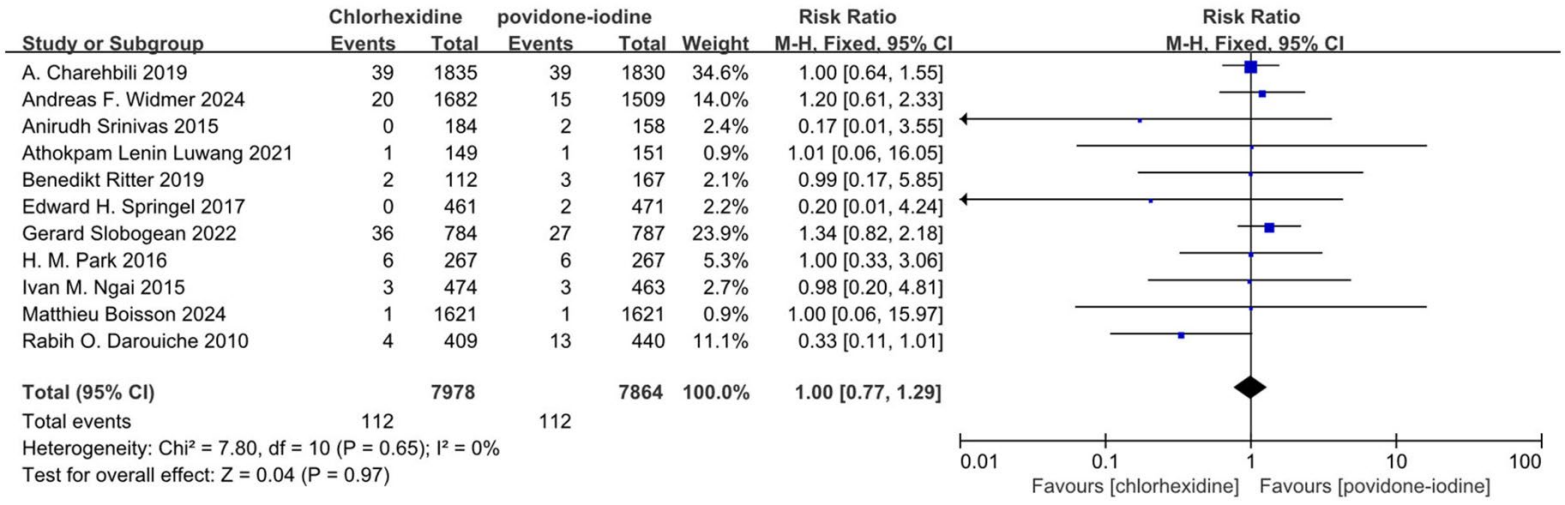
Forest plot of the incidence of deep SSI included in the meta-analysis.

**Figure 5.**
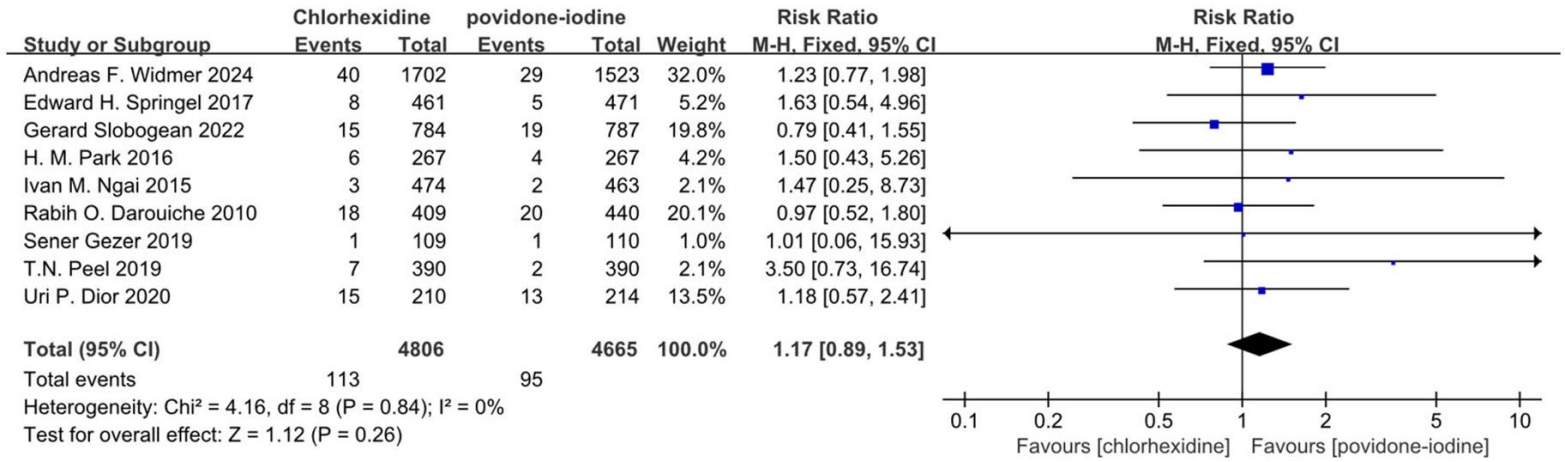
Forest plot of the incidence of organ SSI included in the meta-analysis.

Thirteen studies reported superficial SSI and demonstrated CH was superior to PVI in preventing superficial SSI in Table S2 and Figure 3 (RR: 0.77; 95% CI: 0.64 to 0.92; I^2^ = 17%; *n* = 16,867; *P* = 0.004) [20–22,24,25,27,29,31,32,34,38,39,41]. Eleven studies reported deep SSI and demonstrated CH was not superior to PVI in preventing deep SSI in Table S2 and Figure 4 (RR: 1.00; 95% CI: 0.77 to 1.29; I^2^ = 0%; *n* = 15,842; *P* = 0.97) [20–22,25,27,29,31,34,38,39,41]. Nine studies reported organ SSI and demonstrated CH was not superior to PVI in preventing organ SSI in Table S2 and Figure 5 (RR: 1.17; 95% CI: 0.89 to 1.53; I^2^ = 0%; *n* = 9,471; *P* = 0.26) [22–25,29,31,32,38,41]. The symmetry of the funnel plot and the results of Egger’s test did not reveal any significant evidence of publication bias in Table S8 and Figure S2.

## The secondary outcomes

### Length of hospital stay

Six studies reported the length of hospital stays and demonstrated CH and PVI showed no significant difference in the length of hospital stay in Table S3 and Figure S5 (SMD: 0.04; 95% CI: −0.04 to 0.12; I^2^ = 65%; *n* = 11,959; *P* = 0.30) [20,24,27,33,37,40]. The symmetry of the funnel plot and the results of Egger’s test did not reveal any significant evidence of publication bias in Table S8 and Figure S4.

### Incidence of readmission

Seven studies reported incidence of readmission and demonstrated CH and PVI showed no significant difference in the incidence of readmission in Table S3 and Figure S6 (RR: 1.02; 95% CI: 0.89 to 1.18; I^2^ = 0%; *n* = 11,313; *P* = 0.74) [20,24,26,33,37,38,41]. The symmetry of the funnel plot and the results of Egger’s test did not reveal any significant evidence of publication bias in Table S8 and Figure S3.

### Incidence of reoperation

Three studies reported the incidence of reoperation and demonstrated that CH and PVI showed no significant difference in the incidence of reoperation in Table S3 and Figure S7 (RR: 1.03; 95% CI: 0.89 to 1.19; I^2^ = 0%; *n* = 8,035; *P* = 0.67) [20,25,41]. The symmetry of the funnel plot and the results of Egger’s test did not reveal any significant evidence of publication bias in Table S8 and Figure S3.

### Participant age characteristics incorporated into our meta-analysis

Among the twenty-six included studies, data transformation enabled derivation of mean age with SD from eighteen studies in Table S4 [18,19,21–31,34,35,37–39]. A total of 7,786 patients underwent preoperative disinfection with CH and 7,967 patients underwent preoperative disinfection with PVI. The findings suggest potential heterogeneity in participant age characteristics in the eligible studies in Figure S8 (SMD: −0.02; 95% CI: −0.05 to 0.02; I^2^ = 57%; *n* = 15,753; *P* = 0.32).

### Body mass index (BMI) was incorporated into our meta-analysis

The seven included studies reported BMI, encompassing 2,701 patients who underwent preoperative disinfection with CH and 2,755 patients who underwent preoperative disinfection with PVI in Table S4 [23,26–28,37–39]. The findings suggest potential heterogeneity in participant BMI characteristics in the eligible studies in Figure S9 (SMD: 0.01; 95% CI: −0.04 to 0.07; I^2^ = 63%; *n* = 5,456; *P* = 0.65).

### American Society of Anesthesiologists (ASA) score incorporated into our meta-analysis

The eight included studies reported ASA I, including 8,534 patients who underwent preoperative disinfection with CH and 8,371 patients who underwent preoperative disinfection with PVI in Table S5 and Figure S10 (RR: 1.01; 95% CI: 0.96–1.06; I^2^ = 0%; *n* = 16,905; *P* = 0.74) [21,31,32,36,37,39–41].

The eight included studies reported ASA II, including 8,534 patients who underwent preoperative disinfection with CH and 8,371 patients who underwent preoperative disinfection with PVI in Table S5 and Figure S11 (RR: 1.00; 95% CI: 0.96–1.04; I^2^ = 0%; *n* = 16,905; *P* = 0.93) [21,31,32,36,37,39–41].

The eight included studies reported ASA III, including 8,534 patients who underwent preoperative disinfection with CH and 8,371 patients who underwent preoperative disinfection with PVI in Table S5 and Figure S12 (RR: 0.99; 95% CI: 0.92–1.05; I^2^ = 0%; *n* = 16,905; *P* = 0.67) [21,31,32,36,37,39–41].

The five included studies reported ASA IV, including 6,991 patients who underwent preoperative disinfection with CH and 6,829 patients who underwent preoperative disinfection with PVI in Table S5 and Figure S13 (RR: 1.01; 95% CI: 0.95–1.07; I^2^ = 0%; *n* = 13,820; *P* = 0.80) [21,32,39–41].

The three included studies reported ASA V, including 6,443 patients who underwent preoperative disinfection with CH and 6,255 patients who underwent preoperative disinfection with PVI in Table S5 and Figure S14 (RR: 0.87; 95% CI: 0.49–1.57; I^2^ = 0%; *n* = 12,698; *P* = 0.65) [30,40,41].

### Sex differences in ratios

The seventeen included studies reported the number of males, including 6,100 males who underwent preoperative disinfection with CH and 6,007 males who underwent preoperative disinfection with PVI in Table S6 and Figure S15 (RR: 1.01; 95% CI: 0.99–1.03; I^2^ = 18%; *n* = 24,460; *P* = 0.38) [16,19–22,25,28,30–34,36,37,39–41]. Twenty-three studies reported female sex, with 8,097 and 8,291 patients in the CH and PVI groups in Table S6 and Figure S16 (RR: 0.99; 95% CI: 0.98–1.01; I^2^ = 60%; *n* = 28,449; *P* = 0.46) [19–41]. The findings suggest no potential heterogeneity in the ratio of male differences in the eligible studies. There is potential heterogeneity in the ratio of female differences in the eligible studies.

### Subgroup analysis

Fourteen studies compared CH and PVI for preoperative abdominal disinfection [16,17,21,26–29,31,33,36,38–41]. Five orthopaedic surgery-related studies compared CH and PVI for preoperative skin antisepsis [18,21,25,32,34]. Three cardiothoracic surgeries compared CH and PVI for preoperative skin antisepsis [20,37,41]. The meta-analysis demonstrated CH was not superior to PVI in preventing overall SSI for the abdominal surgery (RR: 0.86; 95% CI: 0.73–1.01; I^2^ = 36%; *n* = 16,541; *P* = 0.06), the orthopaedic surgery (RR: 0.97; 95% CI: 0.67–1.42; I^2^ = 41%; *n* = 6,422; *P* = 0.88), and the cardiothoracic surgery (RR: 1.02; 95% CI: 0.83–1.25; I^2^ = 15%; *n* = 7,559; *P* = 0.86) (Figure S17). Because few secondary outcomes were reported, these outcomes were not analyzed.

After analyzing the age characteristics of the participants, there was a certain degree of heterogeneity in the age characteristics of the participants across different studies. Subgroup analysis results indicate that patients over 50 years of age (RR: 0.84; 95% CI: 0.68–1.03; I^2^ = 18%; *n* = 7,975; *P* = 0.09) [21,22,24,28,30,34,35,37] or under 50 years of age (RR: 0.82; 95% CI: 0.66–1.02; I^2^ = 18%; *n* = 6,669; *P* = 0.08) [18,19,23,25–27,29,31,38,39] who received CH for skin disinfection preoperatively did not have a lower overall postoperative SSI incidence compared to those who received PVI for skin disinfection preoperatively in Figure S18.

### Sensitivity analysis

Since the prevalence of pathogens associated with caesarean section surgery differs from that of other surgical procedures, after excluding the seven articles on caesarean section surgery [23,24,26,27,29,35,38], analysis of the remaining 19 studies revealed that CH was superior to PVI in preventing overall SSI in Figure S19 (RR: 0.91; 95% CI: 0.84–0.99; I^2^ = 35%; *n* = 25,311; *P* = 0.03) [16–22,25,28,30–34,36,37,39–41]. Given that one study received a quality evaluation of ‘some concerns’ [16], we excluded it from the analysis. A sensitivity analysis of the other 25 studies revealed that CH was still superior to PVI in preventing overall SSI in Figure S20 (RR: 0.89; 95% CI: 0.79–0.99; I^2^ = 28%; *n* = 29,300; *P* = 0.03) [17–41].

## Discussion

The pooled evidence from this systematic review and meta-analysis indicates that CH-based preoperative skin preparation is superior to PVI in preventing overall SSI and superficial SSI. In contrast to its effect on the overall SSI, CH was not superior to PVI in preventing deep SSI and organ SSI. For the length of hospital stay, incidence of readmission, and incidence of reoperation, there was no significant difference. Analysis of baseline data for patients included in the study revealed no significant heterogeneity between the CH group and the PVI group in key baseline characteristics such as age, the ratio of males, BMI, and ASA score. The subgroup analysis revealed that CH was not superior to PVI in preventing overall SSI for the abdominal surgery, the orthopaedic surgery, and the cardiothoracic surgery.

A recent systematic review and meta-analysis demonstrated that there is no significant difference in reducing the overall incidence of postoperative SSI between using CH or PVI for vaginal disinfection before hysterectomy [48]. The conclusions drawn from this study are contrary to ours. The review by Trisha et al. included several trials with low methodological quality. So, we suspect the discrepancy may stem from this study’s strict limitation to high-quality RCTs, which were designed to lower the biases commonly found in observational studies.

Our study concluded that CH demonstrates superior disinfection efficacy compared to PVI in preventing overall postoperative SSI. Consistent with our findings, Trisha et al.’s meta-analysis indicated that CH solutions are more effective than PVI solutions in reducing the incidence of SSI [49]. Similarly, a network meta-analysis by Wade et al. revealed that CH is effective in lowering the incidence of postoperative infections following clean surgeries [50]. However, Wade et al. only examined the impact of CH or PVI on the overall postoperative SSI incidence rate, without analyzing their effects on superficial, deep, and organ SSI. This limitation restricts the applicability of their conclusions. This systematic review and meta-analysis compared the incidence of postoperative superficial, deep, and organ SSI following preoperative application of CH versus PVI. Research has revealed that in human skin models, the concentration of chlorhexidine at depths exceeding 300 micrometres has diminished to extremely low levels [51]. Furthermore, the minimum contact time generally recommended for this antiseptic to achieve effective antimicrobial activity is typically 3 to 5 min [52], while its antimicrobial efficacy persists on the skin surface for at least 7 h following a single application [53]. From a biological mechanism perspective, chlorhexidine exhibits rapid onset, prolonged bacteriostatic effects, and potent residual activity. These characteristics likely constitute key factors underpinning its demonstrated advantages in preventing superficial SSI. The meta-analysis concluded that CH is superior to PVI in reducing postoperative superficial SSI. This finding is consistent with that reported by Santos et al. [54]. When comparing the use of CH versus PVI for preventing postoperative organ SSI and deep SSI rates, the results of this study indicate that CH is not superior to PVI.

By analyzing the length of postoperative hospital stays, readmission rates, and reoperation rates, it is possible to indirectly assess the severity of postoperative patient conditions and the burden of care borne by the hospital. The present study revealed that neither preoperative use of CH nor PVI for disinfection shortened postoperative hospital stays, nor did it lower rates of readmission or reoperation. Therefore, it was tentatively concluded that, in the long term, neither CH nor PVI for preoperative skin disinfection of the surgical site reduces the postoperative healthcare burden. However, the studies included in this meta-analysis demonstrated variations in the male-to-female patient ratio, suggesting considerable heterogeneity in baseline population characteristics. This disparity primarily stemmed from seven studies focusing on comparing the disinfection efficacy of CH versus PVI during caesarean sections [23,24,26,27,29,35,38], all of which exclusively enrolled pregnant women, resulting in a highly unbalanced gender distribution. Consequently, the observed heterogeneity has a reasonable clinical and research design rationale. Baseline BMI data from the included studies exhibited considerable heterogeneity, primarily due to two studies reporting mean BMIs and standard deviations significantly higher than those in other investigations. This discrepancy aligns with physiological expectations, as weight gain during pregnancy constitutes a normal physiological process [55], potentially leading to generally elevated BMI measurements.

The subgroup analysis results of this study indicate that for abdominal surgery, there is no difference in the overall postoperative SSI rate regardless of whether CH or PVI is selected for preoperative skin disinfection of the surgical site. Contrary to our conclusion, the systematic review and meta-analysis by Dimitra et al. demonstrated that the efficacy of CH for skin disinfection prior to abdominal surgery is superior to that of PVI [56]. The reason for the differing results may be attributed to the inclusion of newly published RCTs.

To the best of the authors’ knowledge, this study was the first systematic review and meta-analysis comparing the efficacy of PVI versus CH for preoperative skin disinfection in cardiac surgery. Consistent with the findings of this study, Stephen et al. reported that the incidence of postoperative SSI following preoperative application of CH for skin disinfection was comparable to that observed with preoperative application of PVI (11.1% vs 10.5%) [37]. The three studies included in the subgroup analysis all collected and analyzed patient data from developed countries. Therefore, based on the study results, we infer that there is no significant difference in the efficacy of PVI or CH for preoperative skin disinfection prior to cardiac surgery in developed countries.

For orthopaedic surgery, subgroup analysis similarly demonstrated no difference between CH and PVI in preventing postoperative SSI. This finding is consistent with the conclusions of Gerard et al. (7.4% vs 7.5%) [25]. However, contrary to our conclusions, a 2021 meta-analysis by Trisha et al. indicated that PVI may be more suitable for preoperative skin disinfection in orthopaedic surgery [49]. But the studies included in Trisha et al.’s meta-analysis contained several low-quality RCTs, whereas the studies included in this subgroup analysis were all high-quality RCTs, thereby lowering the risk of publication bias.

There is a correlation between the development of SSI and patient age. Studies indicate that within the age range of 17–65 years, the risk of SSI increases by 1.1% annually [57]. Subgroup analysis indicated that while age influences the baseline risk of infection, it does not alter the bactericidal efficacy of disinfectants.

To assess the robustness of this finding, we performed a suite of sensitivity analyses. To evaluate the potential impact of the seven caesarean section studies [23,24,26,27,29,35,38] on the overall results, we conducted a sensitivity analysis. The exclusion of these studies did not materially alter the magnitude or statistical significance of the pooled effect estimate (RR: 0.91; 95% CI: 0.84–0.99; I^2^ = 35%; *n* = 25,311; *P* = 0.03) [16–22,25,28,30–34,36,37,39–41]. When excluding one study deemed to have some concerns” regarding risk-of-bias [16], the analysis indicated that the primary outcome was not predominantly driven by studies of lower methodological quality (RR: 0.89; 95% CI: 0.79–0.99; I^2^ = 28%; *n* = 29,300; *P* = 0.03) [17–41].

A previous study demonstrated that the cost of a postoperative SSI can reach as high as twenty thousand US dollars [58]. Therefore, selecting an effective skin antiseptic prior to surgery to thoroughly disinfect the surgical site becomes particularly crucial in reducing the incidence of postoperative SSI. So CH may represent a cost-effective infection prevention measure by effectively reducing SSI incidence. In resource-limited settings, where other infection control measures (such as antibiotic availability, aseptic technique adherence, and operating room environmental conditions) may be inadequate, selecting a reliable skin antiseptic is particularly crucial for SSI prevention [59]. Conversely, in resource-rich settings, SSI prevention typically relies on a comprehensive approach encompassing multiple measures, including high-grade antibiotics and optimal postoperative care, with the selection of preoperative antiseptics constituting merely one component of this strategy [60]. Therefore, in developing countries where measures to prevent and control surgical site infections following diverse procedures remain relatively limited, the use of preoperative skin antiseptics holds particularly critical practical significance.

This study also has several limitations. First, the manufacturers of PVI and CH included in this study varied across different articles in Table S7, potentially leading to differences in their effective concentrations. Second, when analyzing the application of CH or PVI in cardiac surgery, the number of included studies was too small, and the conclusions drawn may carry a certain risk of bias. Therefore, we hope that future clinical RCTs will focus more on the skin disinfection efficacy of CH or PVI during cardiac surgery. Third, most studies included in our meta-analysis were conducted in developed countries. Nevertheless, postoperative SSI rates differ between developed and developing countries. Consequently, we hope future clinical RCTs will be conducted in developing countries to further supplement and refine our conclusions. Fourth, limitations of this study’s applicability are due to the absence of safety data within the included literature. Inadequate or absent reporting of adverse reactions across all studies prevented a systematic assessment of safety differences between interventions, thereby hindering formal safety analysis. Finally, a key limitation of this meta-analysis is that it cannot evaluate compliance with the intended disinfectant application protocols in the original trials. This is due to a common lack of reporting on critical procedural details in the literature, including whether the recommended contact time and adequate drying time prior to incision were ensured.

## Conclusion

CH demonstrated superiority over PVI in preventing overall SSI and superficial SSI. However, no significant differences were observed between the two agents in deep SSI, organ SSI, or secondary outcomes (length of hospital stay, readmission rates, etc.).

## Supplementary Material

PRISMA.docx

IANN-2025-5681.R1-Revised supplementary material-Clean copy.docx

## Data Availability

All data are secondary and are available in the original published studies. Data produced through meta-analysis will be available with publication (in the manuscript and/or Supplementary Material). Additional information can be supplied by the corresponding author upon reasonable request.
