# Supplementary material for: Comparing the efficacy of chlorhexidine and povidone-iodine for surgical site disinfection: a systematic review and meta-analysis from randomized controlled trials
Source: Ann Med. 2026 Feb 26;58(1):2634531. doi: 10.1080/07853890.2026.2634531 (PMC12951655; doi:10.1080/07853890.2026.2634531)
Supplement: IANN-2025-5681.R1-Revised supplementary material-Clean copy.docx [file IANN_A_2634531_SM8140.docx]

**Supplementary Materials**

**Supplementary Method** Search strategy of the PubMed database in the meta-analysis.

**Supplementary Table 1.** Study inclusion and exclusion criteria.

**Supplementary Table 2.** The incidence of overall SSI, superficial SSI, deep SSI and organ SSI of the participants included in the study.

**Supplementary Table 3.** The secondary outcomes of the participants included in the meta-analysis.

**Supplementary Table 4.** The baseline characteristics of the participants included in the meta-analysis.

**Supplementary Table 5.** The American Society of Anesthesiologists (ASA) score of the participants included in the meta-analysis.

**Supplementary Table 6.** The Sex differences in ratios of the participants included in the meta-analysis.

**Supplementary Table 7.** The general characteristics of studies included in the meta-analysis.

**Supplementary Table 8.** The egger’s publication bias of the primary and secondary outcomes in the meta-analysis.

**Supplementary Figure 1.** Results of the Cochrane Risk-of-Bias Tool, Version2 (Rob2) for Randomized Controlled Trials in the meta-analysis.

**Supplementary Figure 2.** Funnel plot of the incidence of overall SSI, superficial SSI, organ SSI and deep SSI in the meta-analysis.

**Supplementary Figure 3.** Funnel plot of the incidence of readmission, reoperation in the meta-analysis.

**Supplementary Figure 4.** Funnel plot of the length of the hospital stay in the meta-analysis.

**Supplementary Figure 5.** The forest plot of the length of hospital stays included in the meta-analysis.

**Supplementary Figure 6.** The forest plot of the incidence of readmission included in the meta-analysis.

**Supplementary Figure 7.** The forest plot of the incidence of reoperation included in the meta-analysis.

**Supplementary Figure 8.** The forest plot of the participant age characteristics included in the meta-analysis.

**Supplementary Figure 9.** The forest plot of the participant body mass index characteristics included in the meta-analysis.

**Supplementary Figure 10.** The forest plot of the participant ASA Ⅰ score characteristics included in the meta-analysis.

**Supplementary Figure** **11.** The forest plot of the participant ASA Ⅱ score characteristics included in the meta-analysis.

**Supplementary Figure 12.** The forest plot of the participant ASA Ⅲ score characteristics included in the meta-analysis.

**Supplementary Figure 13.** The forest plot of the participant ASA Ⅳ score characteristics included in the meta-analysis.

**Supplementary Figure 14.** The forest plot of the participant ASA Ⅴ score characteristics included in the meta-analysis.

**Supplementary Figure 15.** The forest plot of male participant proportions included in the meta-analysis.

**Supplementary Figure 16.** The forest plot of female participant proportions included in the meta-analysis.

**Supplementary Figure 17.** Subgroup analysis: The forest plot of surgical types included in the meta-analysis of preoperative disinfection.

**Supplementary Figure 18.** Subgroup analysis: The forest plot of age characteristics included in the meta-analysis of preoperative disinfection.

**Supplementary Figure 19**. Sensitivity analysis: Effect estimates after excluding caesarean section studies.

**Supplementary Figure 20**. Sensitivity analysis: Effect estimates after excluding one study with “some concerns”.

**Supplementary Method** Search strategy of the PubMed database in the meta-analysis.

(("chlorhexidine"[Supplementary Concept] OR "chlorhexidine"[All Fields] OR "chlorhexidine acetate"[All Fields] OR "chlorhexidine"[MeSH Terms] OR "chlorhexidine hydrochloride"[All Fields] OR "tubulicid"[All Fields] OR "sebidin a"[All Fields] OR "mk412a"[All Fields])AND("povidone-iodine"[Supplementary Concept] OR "povidone-iodine"[All Fields] OR "povidone-iodine"[MeSH Terms] OR "polyvinylpyrrolidone iodine"[All Fields] OR "pvp iodine"[All Fields] OR "betadine"[All Fields] OR "betaisodona"[All Fields] OR "pharmadine"[All Fields] OR "iodine"[MeSH Terms] OR "iodine"[All Fields])AND("antisep"[All Fields] OR "disinfect"[All Fields] OR "steriliz"[All Fields] OR "antisepsis"[Title/Abstract] OR "disinfection"[Title/Abstract])AND("surgery"[MeSH Subheading] OR "surgery"[All Fields] OR "surgical procedures, operative"[MeSH Terms] OR "general surgery"[MeSH Terms]))

**Supplementary Table 1.** Study inclusion and exclusion criteria.

| **Inclusion Criteria** | **Exclusion Criteria** |
| --- | --- |
| Patients who underwent surgery and received CH or PVI for surgical site disinfection. | Patients undergoing CH or PVI disinfection are not being treated for surgical procedures. |
| Randomized clinical trial. | Case reports, case series, and reviews |
| The reported outcomes including the incidence of overall SSI, superficial SSI, deep SSI, organ SSI, the length of hospital stay, incidence of readmission and incidence of reoperation. | The study's outcome measures did not meet the inclusion criteria. |
| No restrictions on age or gender. | Unable to get full text information. |
| Articles with access to full data and full text. | Non-RCT studies. |

**Supplementary Table 2.** The incidence of overall SSI, superficial SSI, deep SSI and organ SSI of the participants included in the study

| **Author** | **Overall SSI** | **Superficial SSI** | **Deep SSI** | **Organ SSI** |
| --- | --- | --- | --- | --- |
| Andreas F. Widmer | 97/1751 vs 80/1570 | 29/1691 vs 32/1526 | 20/1682 vs 15/1509 | 40/1702 vs 29/1523 |
| Athokpam Lenin Luwang | 8/149 vs 13/151 | 7/149 vs 12/151 | 1/149 vs 1/151 | NA |
| Edward H. Springel | 29/461 vs 33/471 | 21/461 vs 26/471 | 0/461 vs 2/471 | 8/461 vs 5/471 |
| Gerard Slobogean | 58/784 vs 59/787 | 7/784 vs 13/787 | 36/784 vs 27/787 | 15/784 vs 19/787 |
| Ivan M. Ngai | 21/474 vs 21/463 | 15/474 vs 16/463 | 3/474 vs 3/463 | 3/474 vs 2/463 |
| Matthieu Boisson | 65/1621 vs 53/1621 | 38/1621 vs 31/1621 | 1/1621 vs 1/1621 | NA |
| Nicholas F. Rockefeller | 1/61 vs 2 /58 | NA | NA | NA |
| Nisha A. Lakhi | 3/524 vs 12/590 | NA | NA | NA |
| Stephen Ridley Smith | 119/1076 vs 117/1075 | NA | NA | NA |
| Uri P. Dior | 38/210 vs 34/214 | NA | NA | 15/210 vs 13/214 |
| H. M. Park | 15/267 vs 16/267 | 3/267 vs 6/267 | 6/267 vs 6/267 | 6/267 vs 4/267 |
| Rabih O. Darouiche | 39/409 vs 71/440 | 17/409 vs 38/440 | 4/409 vs 13/440 | 18/409 vs 20/440 |
| Adesoji O Ademuyiwa | 322/2858 vs 341/2855 | NA | NA | NA |
| A. R. Berry | 44/453 vs 61/413 | NA | NA | NA |
| Christopher Bibbo | 2/60 vs 5/67 | NA | NA | NA |
| Sarath Chandra Sistla | 14/200 vs 19/200 | NA | NA | NA |
| Diego Abreu | 6/32 vs 4/24 | NA | NA | NA |
| Anirudh Srinivas | 17/184 vs 33/158 | 17/184 vs 31/158 | 0/184 vs 2 /158 | NA |
| Safia Bibi | 12/168 vs 22/220 | NA | NA | NA |
| Benedikt Ritter | 2/112 vs 9/167 | 0/112 vs 5/167 | 2/112 vs 3/167 | NA |
| Veeraya Paocharoen | 5/250 vs 8/250 | NA | NA | NA |
| A. Charehbili | 70/1835 vs 74/1830 | 31/1835 vs 35/1830 | 39/1835 vs 39/1830 | NA |
| T.N. Peel | 12/390 vs 4/390 | 5/390 vs 3/390 | NA | 7/390 vs 2/390 |
| Sener Gezer | 12/109 vs 12/110 | 11/109 vs 11/110 | NA | 1/109 vs 1/110 |
| Fiona S. Reid | 29/141 vs 44/193 | NA | NA | NA |
| Moharam Mohammadian | 5/97 vs 7/96 | NA | NA | NA |

**Supplementary Table 3.** The secondary outcomes of the participants included in the meta-analysis.

| **Author** | **Length of hospital stays** | **Incidence of readmission** | **Incidence of reoperation** |
| --- | --- | --- | --- |
| Andreas F. Widmer | 3.8(2.47) vs 3.76 (1.81) | 41/1751 vs 37/1570 | 103/1075 vs 84/1570 |
| Athokpam Lenin Luwang | NA | NA | NA |
| Edward H. Springel | NA | 5/461 vs 9/471 | NA |
| Gerard Slobogean | NA | NA | 115/734 vs 118/738 |
| Ivan M. Ngai | NA | NA | NA |
| Matthieu Boisson | 10 (4.44) vs 10 (4.44) | 227/1621 vs 211/1621 | 125/1621 vs 121/1621 |
| Nicholas F. Rockefeller | NA | NA | NA |
| Nisha A. Lakhi | NA | 3/524 vs 3/590 | NA |
| Stephen Ridley Smith | 4.2 (6.2) vs 4.4 (7.2) | 28/1076 vs 34/1075 | NA |
| Uri P. Dior | NA | NA | NA |
| H. M. Park | NA | NA | NA |
| Rabih O. Darouiche | NA | NA | NA |
| Adesoji O Ademuyiwa | 6.4 (4.4) vs 6.4 (4.9) | NA | NA |
| A. R. Berry | NA | NA | NA |
| Christopher Bibbo | NA | NA | NA |
| Sarath Chandra Sistla | NA | NA | NA |
| Diego Abreu | NA | NA | NA |
| Anirudh Srinivas | NA | NA | NA |
| Safia Bibi | NA | NA | NA |
| Benedikt Ritter | NA | NA | NA |
| Veeraya Paocharoen | NA | NA | NA |
| A. Charehbili | NA | NA | NA |
| T.N. Peel | NA | NA | NA |
| Sener Gezer | 5 (2.2) vs 4 (2.2) | 10/109 vs 14/110 | NA |
| Fiona S. Reid | 8.7 (8.9) vs 7.4 (5) | 15/141 vs 14/193 | NA |
| Moharam Mohammadian | NA | NA | NA |

**Supplementary Table 4.** The baseline characteristics of the participants included in the meta-analysis.

| **Author** | **Country** | **Number (CH vs PVI)** | **Operation of surgery** | **Center** | **Age (mean, SD)** | **BMI (mean, SD)** |
| --- | --- | --- | --- | --- | --- | --- |
| Andreas et al., 2024 | Switzerland | 1751 vs 1570 | Abdominal  Cardiac | Multicenter | NA | NA |
| Athokpam et al., 2021 | India | 149 vs 151 | Cesarean Delivery | Single | 28.17 (4.75) vs 27.85 (4.15) | 25.03 (4.14) vs 25.48 (4.28) |
| Edward et al., 2017 | America | 461 vs 471 | Cesarean Antisepsis | Single | 28.0 (6.67) vs 28.0 (5.93) | 35 (8.89) vs 36 (9.63) |
| Gerard et al., 2022 | Multi-countries | 784 vs 787 | Open Fractures | Multicenter | 44.5 (17.9) vs 45.2 (18.2) | NA |
| Ivan et al., 2015 | America | 474 vs 463 | Cesarean Delivery | Multicenter | 30.3 (5.7) vs 29.9 (6.0) | NA |
| Matthieu et al., 2024 | France | 1621 vs 1621 | Cardiac | Multicenter | NA | NA |
| Nicholas et al., 2022 | America | 61 vs 58 | Urogynecology | Single | 58.0 (13.0) vs 57.0 (12.0) | NA |
| Nisha et al., 2019 | America | 524 vs 590 | Cesarean Delivery | Single | 32.49(5.56) vs 32.61(5.22) | 32.48 (6.42) vs 32.99 (6.63) |
| Stephen et al., 2022 | Australia | 1076 vs 1075 | Incisional | Multicenter | 56.0 (17.0) vs 57.0 (17.0) | 31 (7) vs 30 (7) |
| Uri et al., 2020 | Australia | 210 vs 214 | Gynecological Laparoscopic | Single | 35.5 (10.3) vs 36.1(10.8) | 26.3 (6.1) vs 26.6 (6.7) |
| H. M. Park et al., 2016 | South Korea | 267 vs 267 | Abdominal | Single | NA | NA |
| Rabih et al., 2010 | America | 409 vs 440 | Clean-Contaminated | Multicenter | 53.3 (14.6) vs 52.9 (14.2) | NA |
| Adesoji et al., 2021 | Multi-countries | 2858 vs 2855 | Abdominal | Multicenter | NA | NA |
| A. R. Berry et al., 1982 | England | 453 vs 413 | General | Single | NA | NA |
| Christopher et al., 2005 | America | 60 vs 67 | Foot and Ankle | Single | 48 (15.8) vs 45 (17.3) | NA |
| Sarath et al., 2010 | India | 200 vs 200 | Open Hernia Repair | Single | NA | NA |
| Diego et al., 2014 | Brazil | 32 vs 24 | Benign Prostatic Hyperplasia | Single | NA | NA |
| Anirudh et al., 2015 | India | 184 vs 158 | Upper Abdominal | Single | 44.7 (13.7) vs 47.4 (13.1) | 23.09 (2.27) vs 23.12 (2.23) |
| Safia et al., 2015 | Pakistan | 168 vs 220 | Clean or Clean -Contaminated | Multicenter | 40.4 (13.9) vs 41.3 (15.5) | NA |
| Benedikt et al., 2019 | Germany | 112 vs 167 | Lower Limb Trauma | Single | 51.1 (1.6) vs 50.5 (1.3) | NA |
| Veeraya et al., 2009 | Thailand | 250 vs 250 | Clean, Clean- Contaminated, Contaminated | Single | 50.5 (15) vs 56.2 (14.8) | NA |
| Charehbili et al., 2019 | Netherlands | 1835 vs 1830 | Breast, Vascular, Colorectal, Gallbladder or Orthopedic | Multicenter | 65 (18.8) vs 65 (20) | NA |
| T.N. Peel et al., 2019 | Australia | 390 vs 390 | Elective Arthroplasty | Single | NA | NA |
| Sener et al., 2019 | Turkey | 109 vs 110 | malignant and premalignant gynecologic | Single | 53.2 (12.7) vs 53 (12) | NA |
| Fiona S. Reid et al., 2022 | Australia | 141 vs 193 | Colorectal | Single | NA | NA |
| Moharam Mohammadian et al., 2024 | Iran | 97 vs 96 | Elective Abdominal | Single | 55.3 (10.9) vs 54.7 (10.9) | 28.5 (5.9) vs 28.4 (6.9) |

SD, standard deviation.

**Supplementary Table 5.** The American Society of Anesthesiologists (ASA) score of the participants included in the meta-analysis.

| **Author** | **ASA Ⅰ** | **ASA Ⅱ** | **ASA Ⅲ** | **ASA Ⅳ** | **ASA Ⅴ** |
| --- | --- | --- | --- | --- | --- |
| Andreas F. Widmer | 21/1750 vs 26/1570 | 229/1750 vs 216/1570 | 532/1750 vs 469/1570 | 961/1750 vs 848/1570 | 7/1750 vs 11/1570 |
| Athokpam Lenin Luwang | NA | NA | NA | NA | NA |
| Edward H. Springel | NA | NA | NA | NA | NA |
| Gerard Slobogean | NA | NA | NA | NA | NA |
| Ivan M. Ngai | NA | NA | NA | NA | NA |
| Matthieu Boisson | NA | NA | NA | NA | NA |
| Nicholas F. Rockefeller | NA | NA | NA | NA | NA |
| Nisha A. Lakhi | NA | NA | NA | NA | NA |
| Stephen Ridley Smith | 13/1076 vs 12/1075 | 50/1076 vs 51/1075 | 34/1076 vs 34/1075 | 3/1076 vs 3/1075 | NA |
| Uri P. Dior | NA | NA | NA | NA | NA |
| H. M. Park | 103/267 vs 108/267 | 157/267 vs 149/267 | 7/267 vs 10/267 | NA | NA |
| Rabih O. Darouiche | NA | NA | NA | NA | NA |
| Adesoji O Ademuyiwa | 1275/2858 vs 1262/2855 | 1068/2858 vs 1050/2855 | 379/2858 vs 408/2855 | 84/2858 vs 89/2855 | 13/2858 vs 10/2855 |
| A. R. Berry | NA | NA | NA | NA | NA |
| Christopher Bibbo | NA | NA | NA | NA | NA |
| Sarath Chandra Sistla | 115/200 vs 94/200 | 81/200 vs 101/200 | 4/200 vs 5/200 | NA | NA |
| Diego Abreu | NA | NA | NA | NA | NA |
| Anirudh Srinivas | 36/158 vs 48/184 | 94/158 vs 110/184 | 24/158 vs 24/184 | 4/158 vs 3/184 | 0/158 vs 0/184 |
| Safia Bibi | NA | NA | NA | NA | NA |
| Benedikt Ritter | NA | NA | NA | NA | NA |
| Veeraya Paocharoen | NA | NA | NA | NA | NA |
| A. Charehbili | 399/1835 vs 397/1830 | 1137/1835 vs 1120/1830 | 266/1835 vs 272/1830 | 19/1835 vs 25/1830 | 1/1835 vs 2/1830 |
| T.N. Peel | 17 /390 vs 17/390 | 187/390 vs 198/390 | 176/390 vs 168/390 | 10/390 vs 7/390 | NA |
| Sener Gezer | NA | NA | NA | NA | NA |
| Fiona S. Reid | NA | NA | NA | NA | NA |
| Moharam Mohammadian | NA | NA | NA | NA | NA |

**Supplementary Table 6.** The Sex differences in ratios of the participants included in the meta-analysis.

| **Author** | **Male (% CH vs PVI)** | **Female (% CH vs PVI)** |
| --- | --- | --- |
| Andreas F. Widmer | 66.1% vs 67.3% | 33.9% vs 32.7% |
| Athokpam Lenin Luwang | 0% vs 0% | 100% vs 100% |
| Edward H. Springel | 0% vs 0% | 100% vs 100% |
| Gerard Slobogean | 63% vs 61% | 37% vs 39% |
| Ivan M. Ngai | 0% vs 0% | 100% vs 100% |
| Matthieu Boisson | 78.7% vs 76.9% | 21.3% vs 22.1% |
| Nicholas F. Rockefeller | 0% vs 0% | 100% vs 100% |
| Nisha A. Lakhi | 0% vs 0% | 100% vs 100% |
| Stephen Ridley Smith | 46% vs 45% | 54% vs 55% |
| Uri P. Dior | 0% vs 0% | 100% vs 100% |
| H. M. Park | 73.8% vs 66.7% | 26.2% vs 33.3% |
| Rabih O. Darouiche | 58.9% vs 55.9% | 41.1% vs 44.1% |
| Adesoji O Ademuyiwa | 38.2% vs 38.6% | 61.8% vs 61.4% |
| A. R. Berry | NA | NA |
| Christopher Bibbo | NA | NA |
| Sarath Chandra Sistla | 96.5% vs 99% | 3.5% vs 1% |
| Diego Abreu | 100% vs 100% | 0% vs 0% |
| Anirudh Srinivas | 38 % vs 38 % | 62% vs 62% |
| Safia Bibi | 37.5% vs 40.4% | 62.5% vs 59.6% |
| Benedikt Ritter | 48.2% vs 43.7% | 51.8% vs 56.3% |
| Veeraya Paocharoen | 63.6% vs 55.2% | 36.4% vs 44.8% |
| A. Charehbili | 28.2% vs 28.7% | 71.8% vs 71.3% |
| T.N. Peel | 38.5% vs 35.5% | 61.5% vs 64.5% |
| Sener Gezer | 0% vs 0% | 100% vs 100% |
| Fiona S. Reid | 39% vs 44% | 61% vs 56% |
| Moharam Mohammadian | 47.4% vs 44.7% | 52.6% vs 55.3% |

**Supplementary Table 7.** The general characteristics of studies included in the meta-analysis.

| **Author** | **Criterion of SSI** | **Follow-up time** | **Type and concentration of disinfectants** | **Disinfectant manufacturer** |
| --- | --- | --- | --- | --- |
| Andreas et al,2024 | CDC | 30 days or 1 year | 2% CH-Alc  10% PVI-Alc | B. Braun |
| Athokpam et al., 2021 | CDC | 30 days | 2% CH-Alc  10% PVI-Alc | NA |
| Edward et al., 2017 | CDC | 30 days | 2% CH-Alc  (0.75%, 1%) PVI-Aqu | NA |
| Gerard et al., 2022 | CDC | 30 or 90 days | 4% CH-Aqu  10% PVI-Aqu | Medline and Others |
| Ivan et al., 2015 | CDC | 30 days | CH-Alc  PVI-Alc,  Concentration unknown | NA |
| Matthieu et al., 2024 | CDC | 30 or 90 days | 2% CH-Alc  5% PVI-Alc | Becton Dickinson (CH)  Viatris Santé (PVI) |
| Nicholas et al., 2022 | CDC | 2 or 6 weeks | 2% CH-Aqu  10% PVI-Aqu | NA |
| Nisha et al., 2019 | NA | 14 days | 4% CH-Alc  10% PVI-Alc | Becton (CH)  Aplicare (PVI) |
| Stephen et al., 2022 | CDC | 30 or 90 days | (2%,0.5%) CH-Alc  10%PVI-Alc | Perrigo (CH)  Pfizer (PVI) |
| Uri et al., 2020 | CDC | 1 or 4 weeks | (2%,0.015%) CH-Alc  10% PVI-Alc | Baxter (CH)  Pfizer (PVI) |
| H. M. Park et al., 2016 | CDC | 30 days | (4%, 2%) CH-Aqu (7.5%, 10%) PVI-Aqu | Firson |
| Rabih et al., 2010 | CDC | 30 days | 2% CH-Alc  10% PVI-Aqu | Cardinal Health |
| Adesoji et al., 2021 | CDC | 30 days | 2% CH-Alc  10% PVI-Aqu | NA |
| A. R. Berry et al., 1982 | NA | 4 days | 0.5% CH-Alc  10% PVI-Alc | NA |
| Christopher et al., 2005 | NA | NA | 4% CH-Alc  (7.5%,10%) PVI-Alc | NA |
| Sarath et al., 2010 | CDC | 30 days | 2.5% CH-Alc  10% PVI-Alc | NA |
| Diego et al., 2014 | CDC | NA | 0.5% CH-Alc  0.5% PVI-Alc | NA |
| Anirudh et al., 2015 | CDC | 30 days | 0.5% CH-Alc  5% PVI-Alc | NA |
| Safia et al., 2015 | CDC | 30 days | 2% CH-Alc  10% PVI-Alc | NA |
| Benedikt et al., 2019 | CDC | 6 months | 2% CH-Alc  1% PVI-Alc | CareFusion (CH)  B. Braun (PVI) |
| Veeraya et al., 2009 | NA | 1 months | 4% CH-Alc  PVI unknown | NA |
| Charehbili et al., 2019 | CDC | 30 days | 0.5% CH-Alc  1% PVI-Alc | NA |
| T.N. Peel et al., 2019 | CDC | 30,90 days or 1 year | 0.5% CH-Alc  1% PVI-Alc | Orion Laboratories Pty Ltd |
| Sener et al., 2019 | CDC | 30 days | 4% CH-Alc  10% PVI-Alc | Kimpa Drugs (CH)  Necm Chemistry (PVI) |
| Fiona S. Reid et al., 2022 | CDC | 30 days | 2% CH-Alc  PVI-Alc unknown | NA |
| Moharam Mohammadian et al., 2024 | NA | 1 month | 1% CH-Alc  10% PVI-Alc | NA |

CDC, Centers for Disease Control; CH, chlorhexidine; PVI, povidone-iodine; Alc, alcohol; Aqu, aqueous;

**Supplementary Table 8.** The egger’s publication bias of the primary and secondary outcomes in the meta-analysis.

| **Std _Eff**  **Slope bias** | **Coef.** | **Std. Err.** | **t** | **P>\|t\|** | **[95% Conf. Interval]** |
| --- | --- | --- | --- | --- | --- |
| Overall SSI | .0283863 | .0738591 | 0.38 | 0.704 | -.1240514 .1808239 |
|  | -.6385367 | .3517912 | -1.82 | 0.082 | -1.364598 .0875246 |
| Superficial SSI | .0005401 | .2594578 | 0.00 | 0.998 | -.5705586 .5715669 |
|  | -.7743956 | .7694993 | -1.01 | 0.336 | -2.468052 .919261 |
| Organ SSI | -.115849 | .2092137 | -0.55 | 0.597 | -.6105609 .3788629 |
|  | .6887092 | .5023184 | 1.37 | 0.213 | -.499085 1.876503 |
| Deep SSI | .2954385 | .1755797 | 1.68 | 0.127 | -.1017504 .6926274 |
|  | -.7600368 | .39338695 | -1.93 | 0.086 | -1.651031 .130958 |
| Readmission | .1018842 | .092747 | 1.10 | 0.322 | -.1365295 .3402978 |
|  | -.5053973 | .4688836 | -1.08 | 0.330 | -1.710701 .6999062 |
| Reoperation | -.5020399 | .2558564 | -1.96 | 0.300 | -3.753004 2.748924 |
|  | 4.148244 | 1.995343 | 2.08 | 0.285 | -21.20499 29.50148 |
| Hospital stay | -.0753926 | .0448662 | -1.68 | 0.168 | -.1999611 .0491758 |
|  | 2.184864 | 1.00088 | 2.18 | 0.094 | -.5940258 4.963753 |


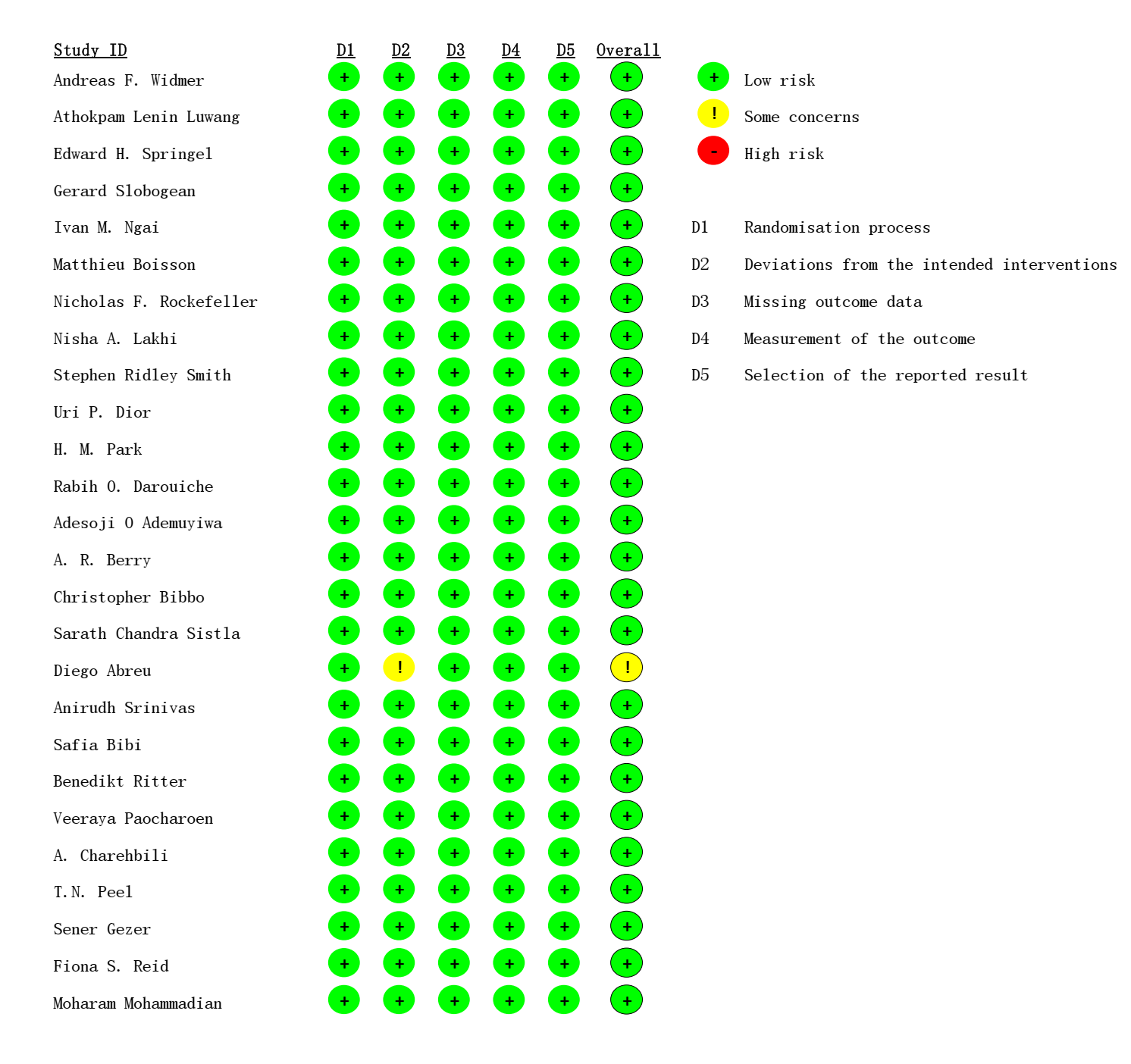


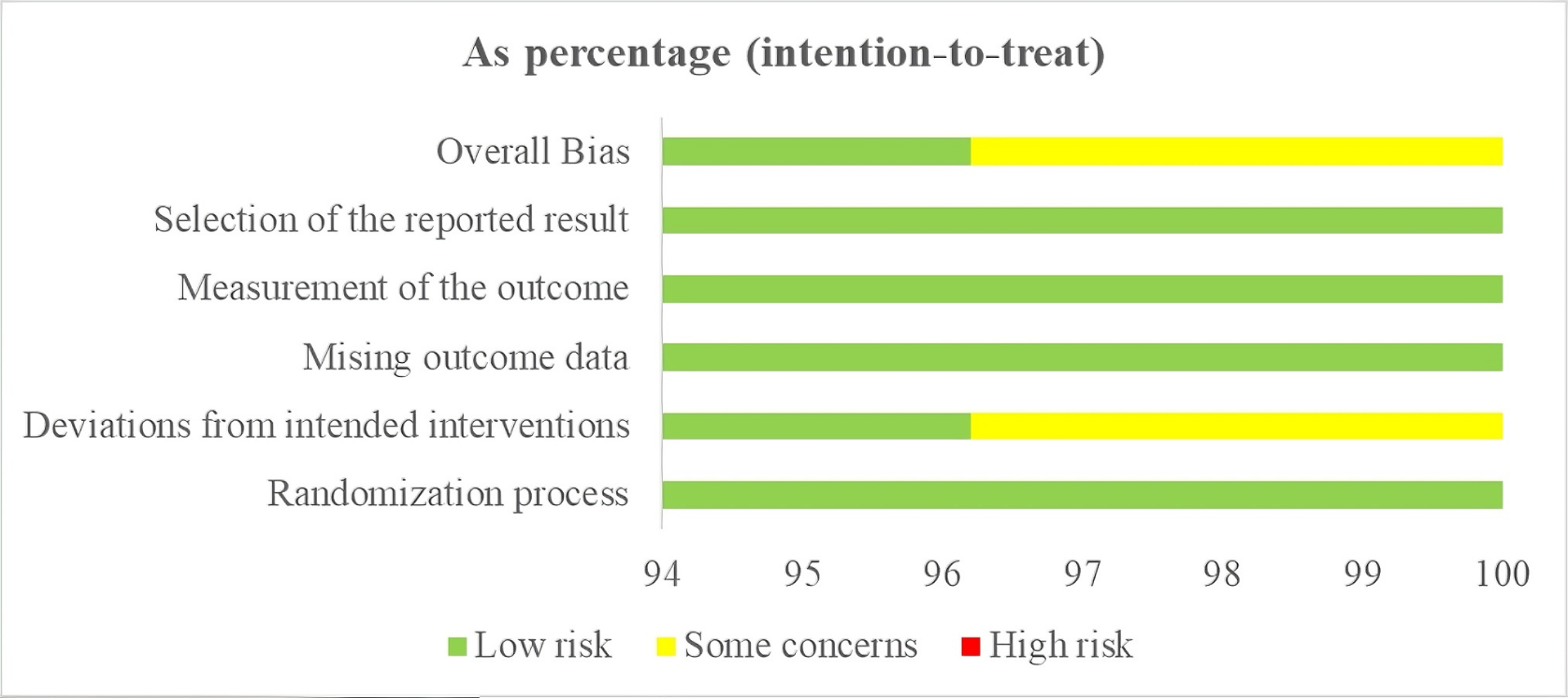


**Supplementary Figure 1.** Results of the Cochrane Risk-of-Bias Tool, Version 2 (Rob2) for randomized controlled trials in the meta-analysis


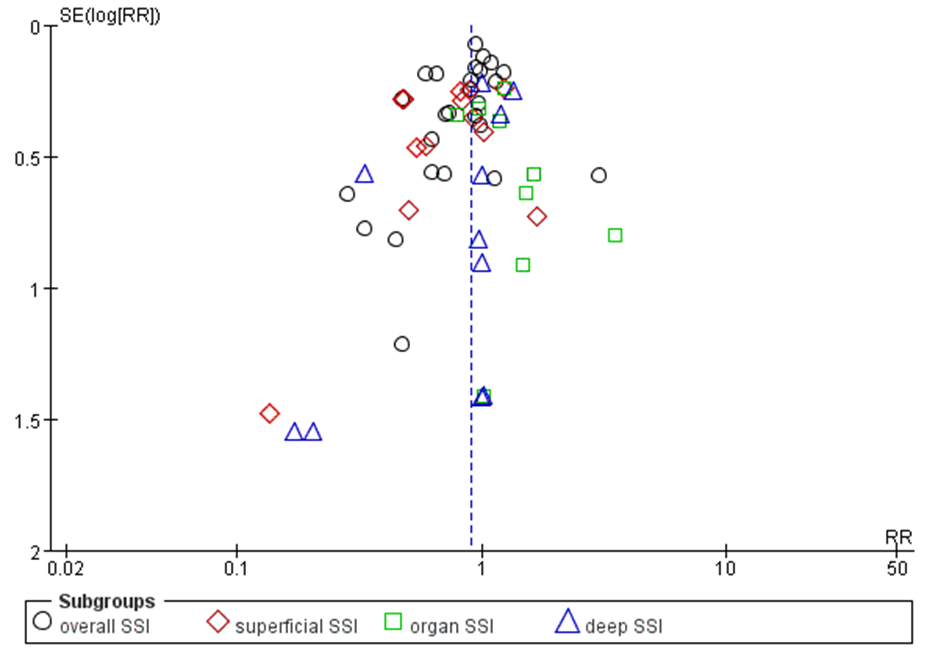


**Supplementary Figure 2.** Funnel plot of the incidence of overall SSI, superficial SSI, organ SSI and deep SSI in the meta-analysis. RR risk ratio; SSI surgical site infection.


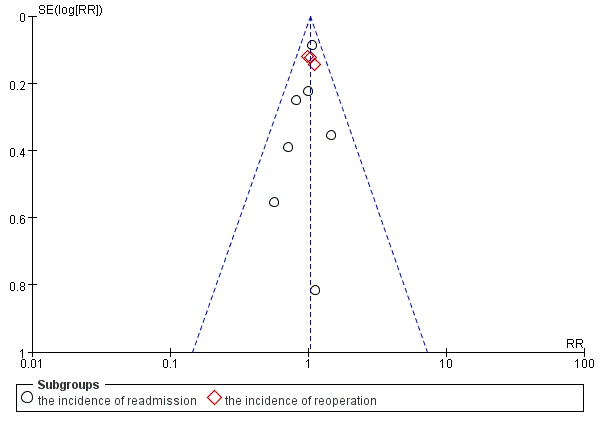


**Supplementary Figure 3.** Funnel plot of the incidence of readmission, reoperation in the meta-analysis. RR, risk ratio.


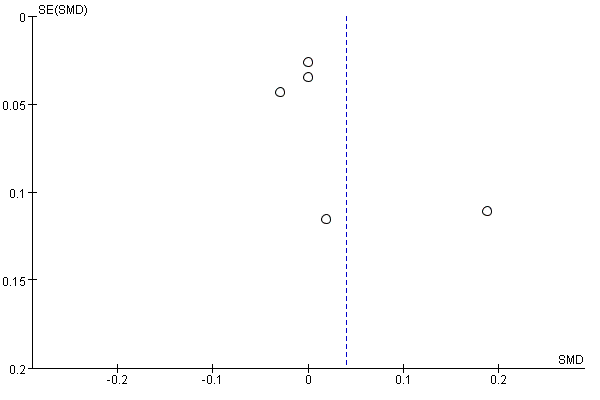


**Supplementary Figure 4.** Funnel plot of the length of the hospital stay in the meta-analysis. SMD, standardized mean differences.


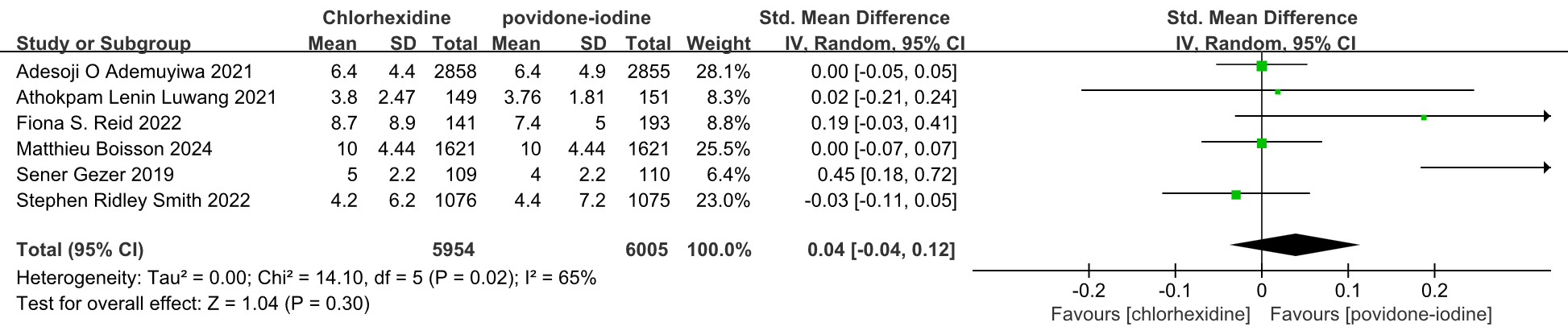


**Supplementary Figure 5**. The forest plot of the length of hospital stays included in the meta-analysis. SD, standard deviation; CI, confidence interval.


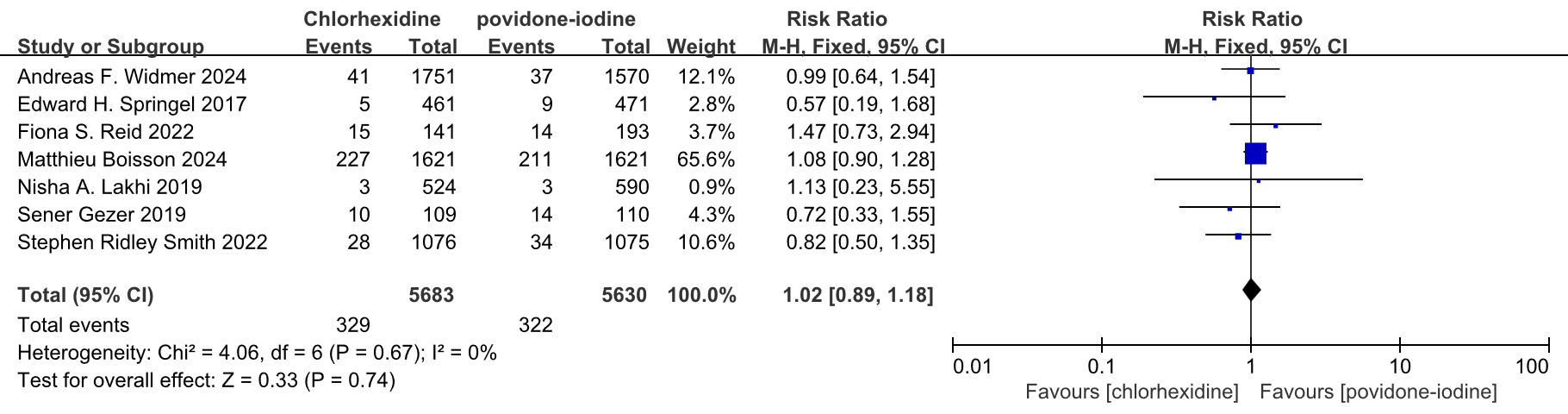


**Supplementary Figure 6.** The forest plot of the incidence of readmission included in the meta-analysis.


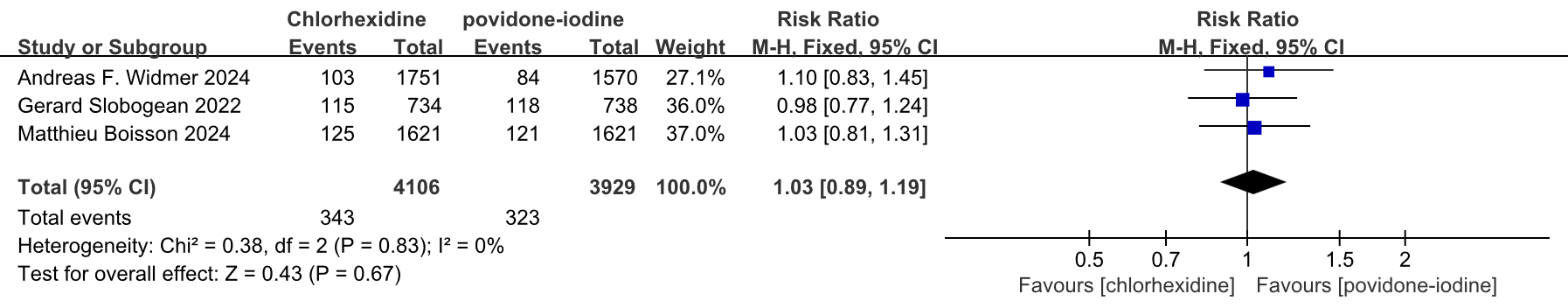


**Supplementary Figure 7**. The forest plot of the incidence of reoperation included in the meta-analysis.


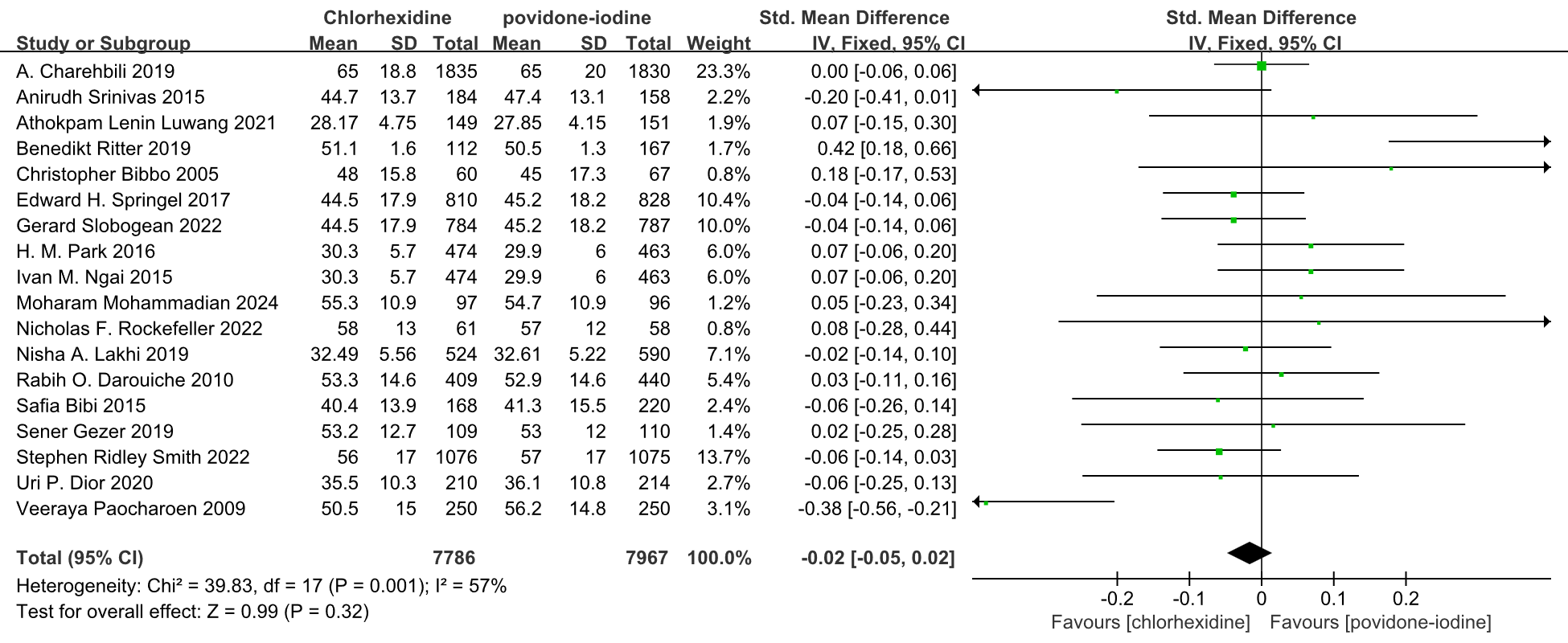


**Supplementary Figure 8**. The forest plot of the participant age characteristics included in the meta-analysis.


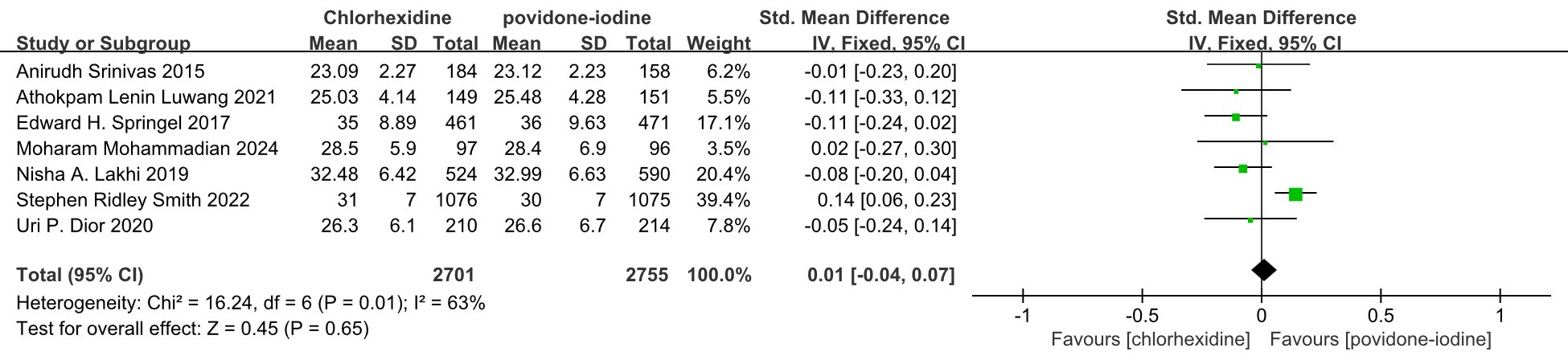


**Supplementary Figure 9.** The forest plot of the participant body mass index characteristics included in the meta-analysis.


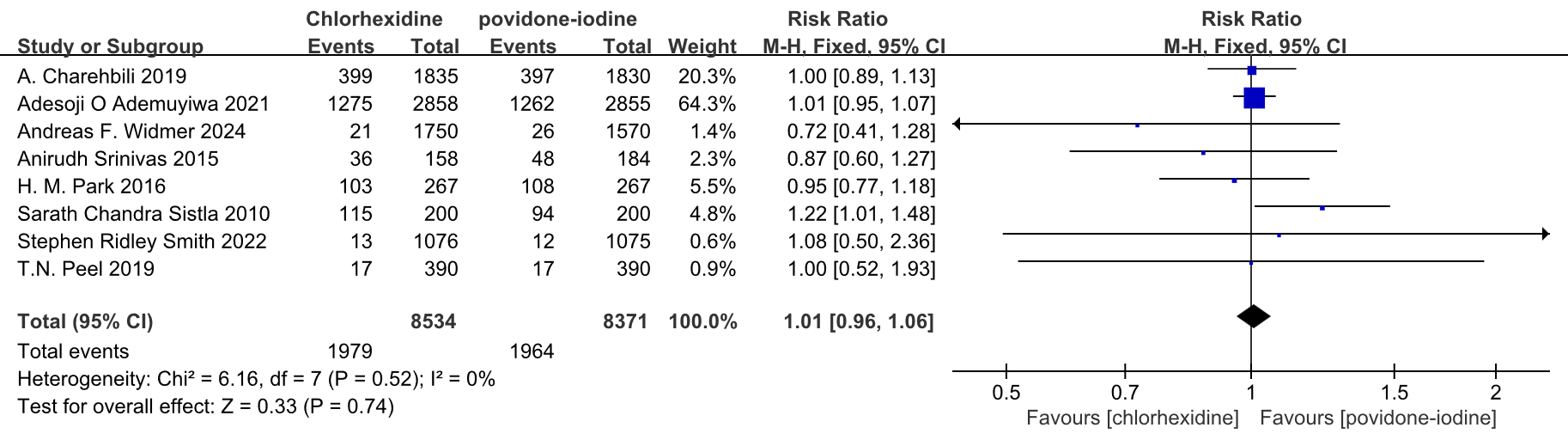


**Supplementary Figure 10**. The forest plot of the participant ASA Ⅰ score characteristics included in the meta-analysis. ASA, American Society of Anesthesiologists.


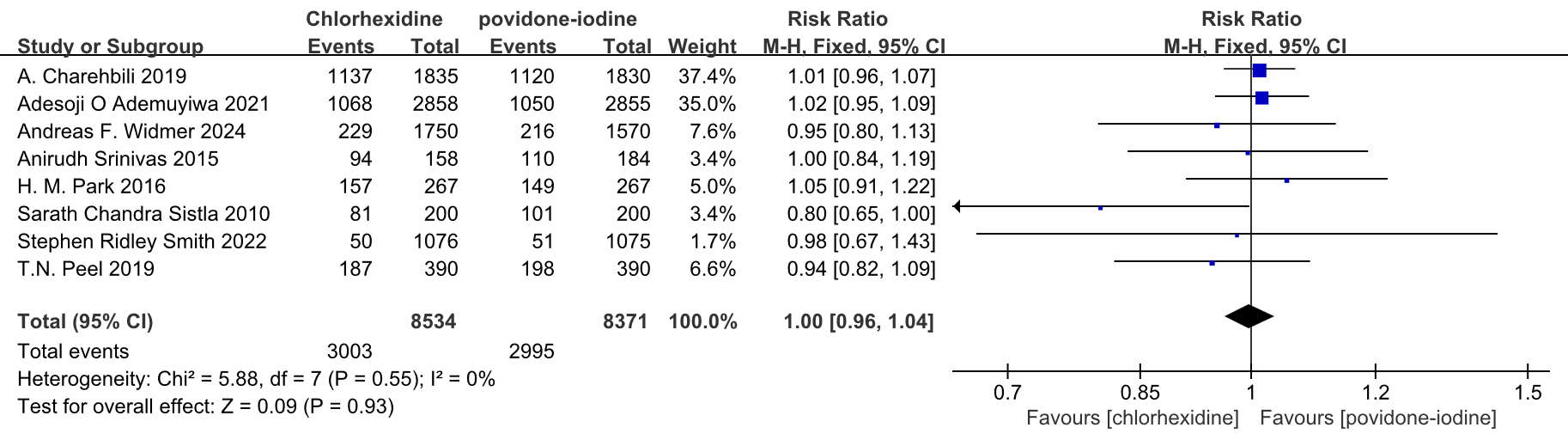


**Supplementary Figure 11**. The forest plot of the participant ASA Ⅱ score characteristics included in the meta-analysis.


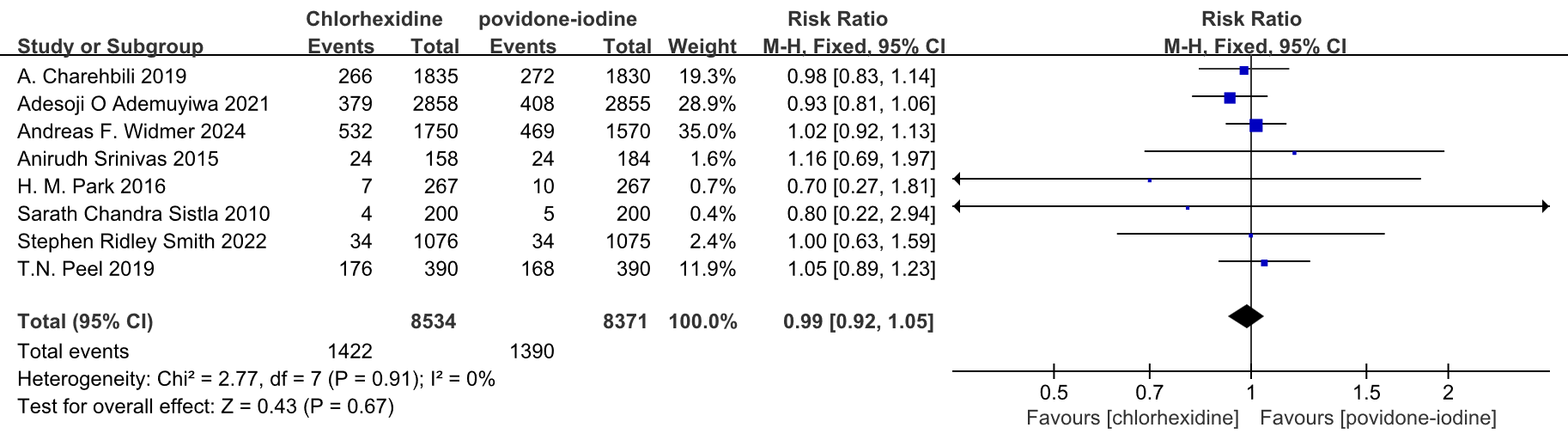


**Supplementary Figure 12**. The forest plot of the participant ASA Ⅲ score characteristics included in the meta-analysis.


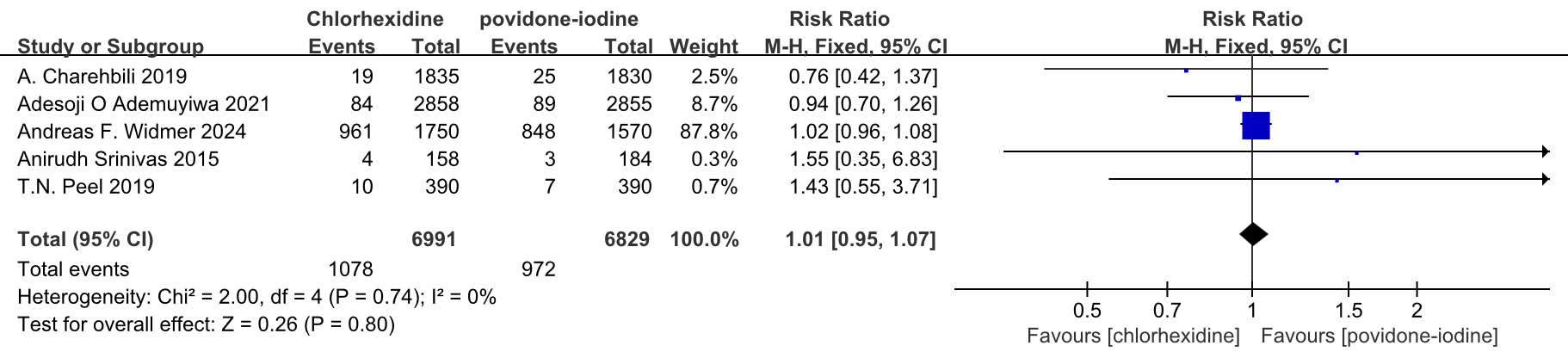


**Supplementary Figure 13**. The forest plot of the participant ASA Ⅳ score characteristics included in the meta-analysis.


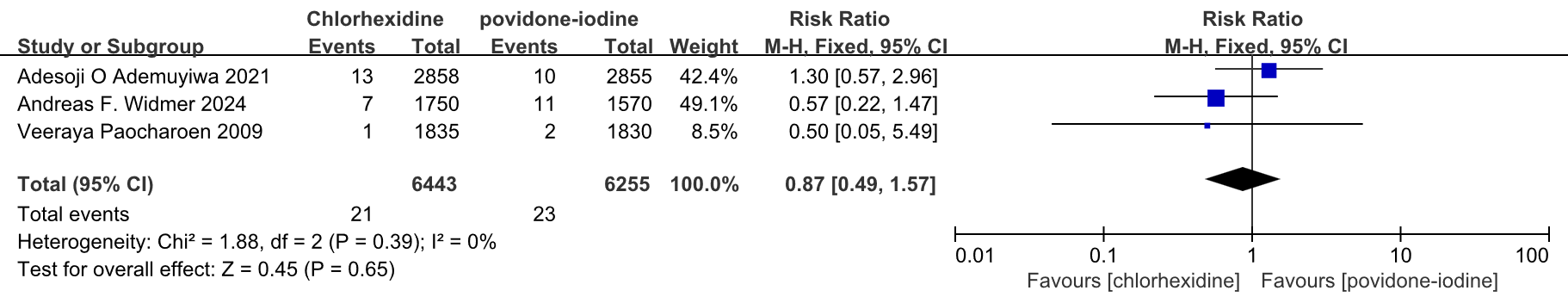


**Supplementary Figure 14**. The forest plot of the participant ASA Ⅴ score characteristics included in the meta-analysis.


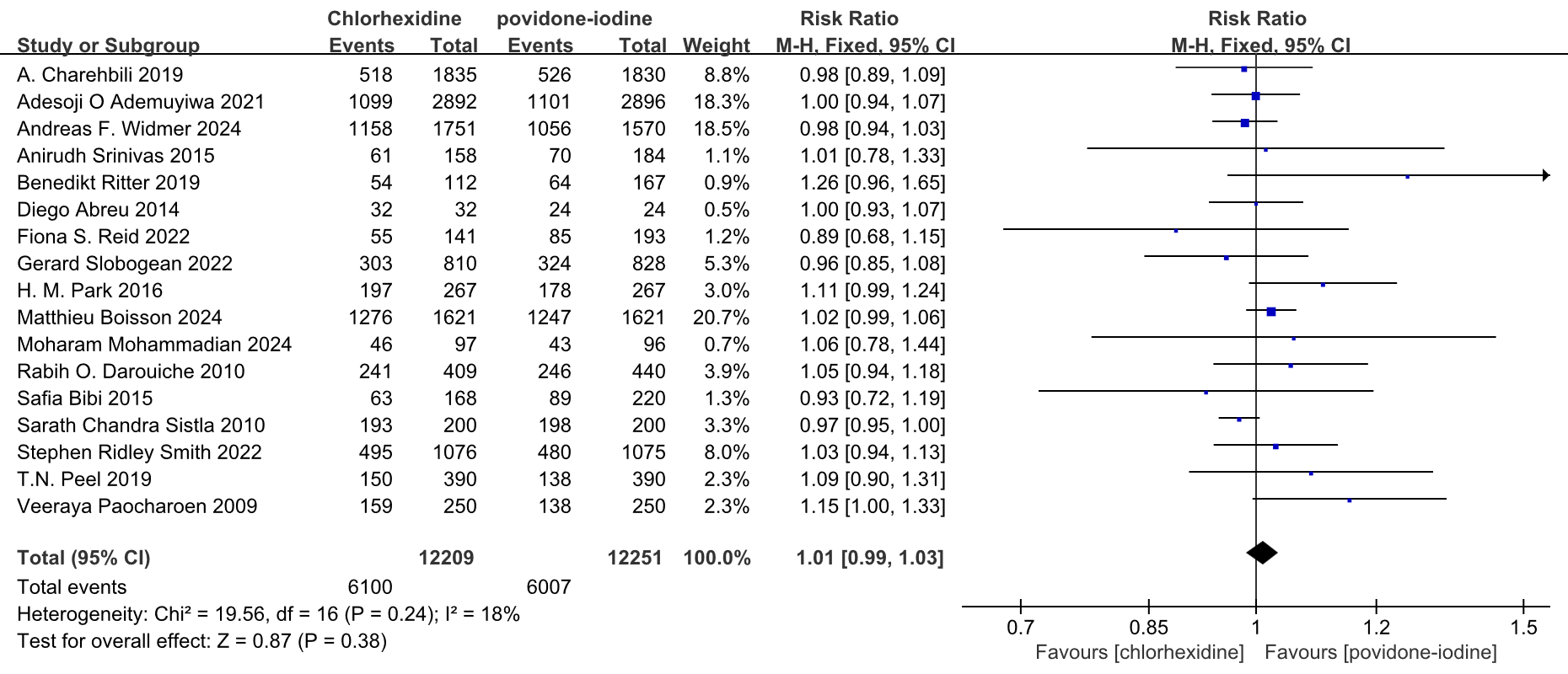


**Supplementary** **Figure 15**. The forest plot of male participant proportions included in the meta-analysis.


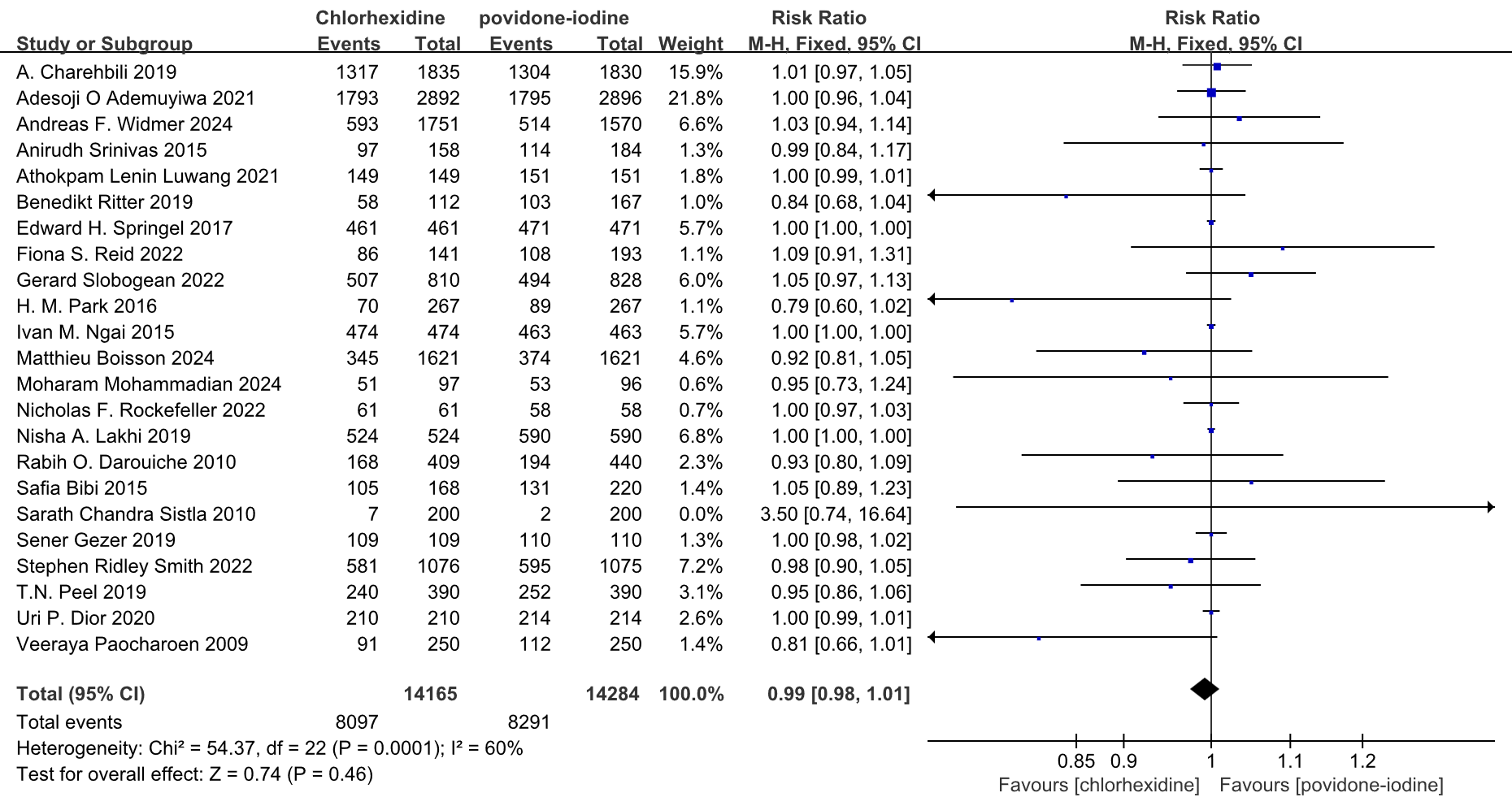


**Supplementary Figure 16**. The forest plot of female participant proportions included in the meta-analysis.


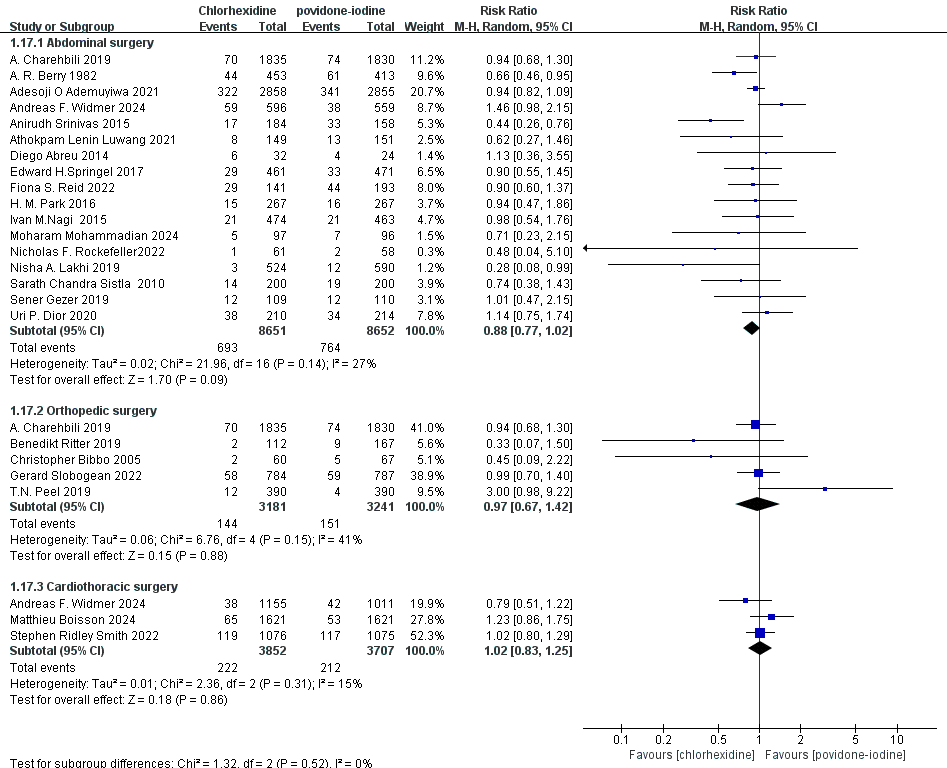


**Supplementary Figure 17.** Subgroup analysis: The forest plot of surgical types included in the meta-analysis of preoperative disinfection. 1.17.1 abdominal surgery; 1.17.2 orthopedic surgery; 1.17.3 cardiothoracic surgery.


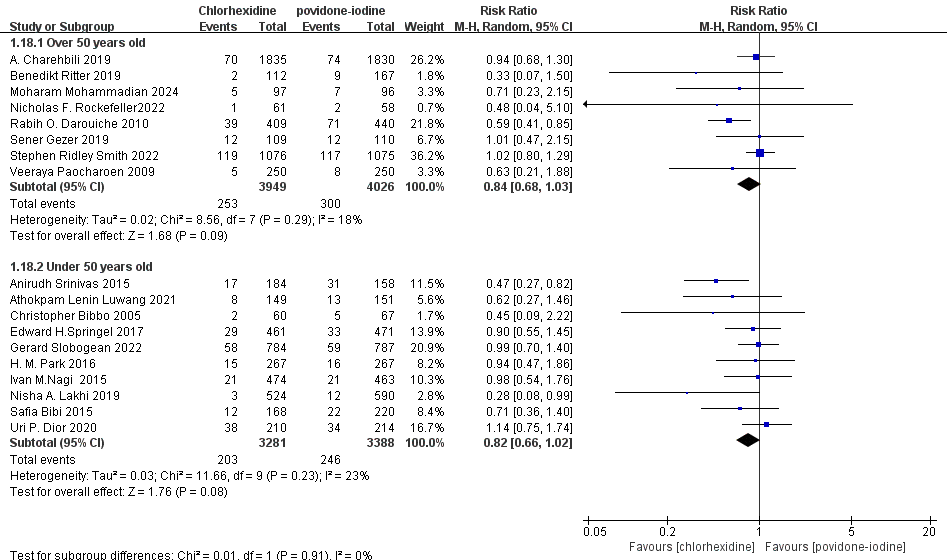


**Supplementary Figure 18.** Subgroup analysis: The forest plot of age characteristics included in the meta-analysis of preoperative disinfection. 1.18.1 over 50 years old; 1.18.2 under 50 years old.


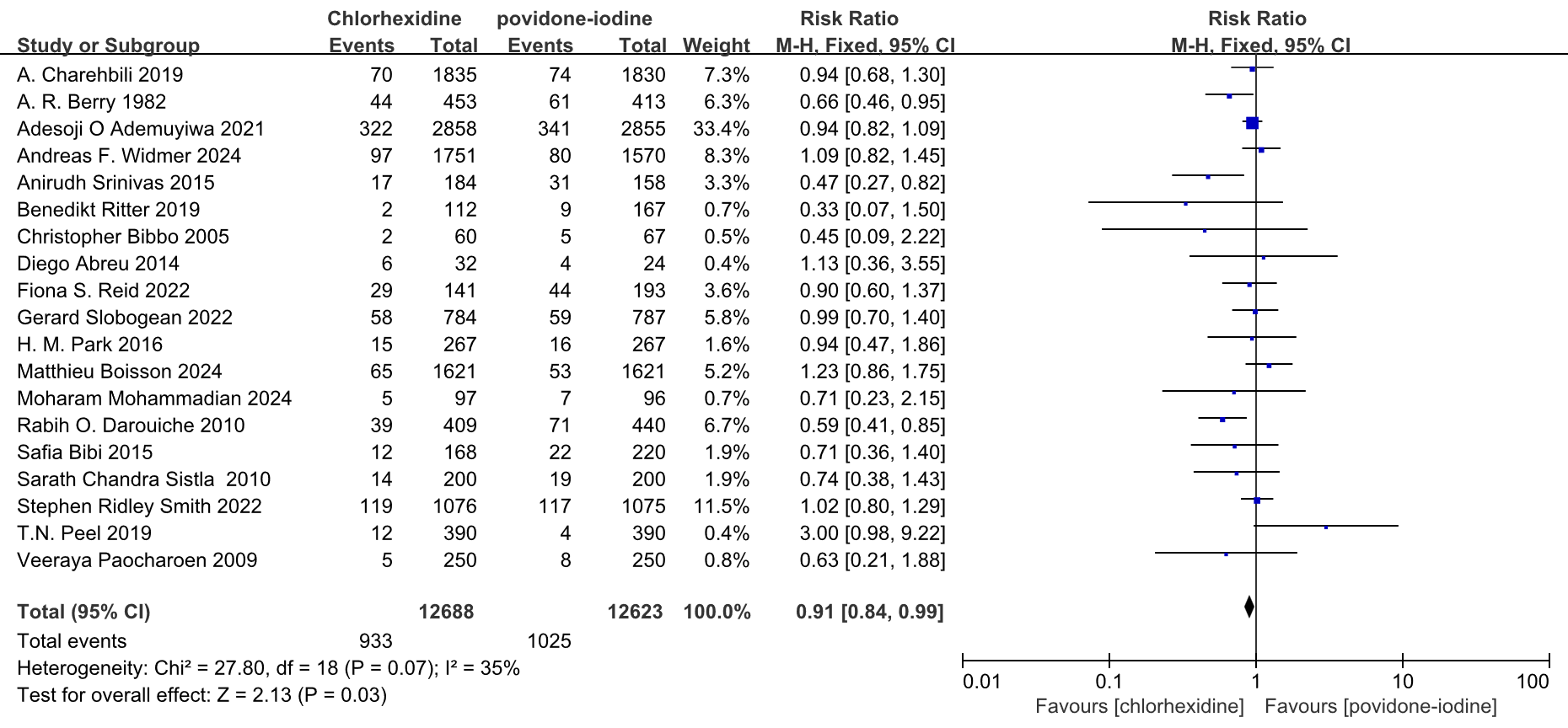


**Supplementary Figure 19**. Sensitivity analysis: Effect estimates after excluding caesarean section studies.


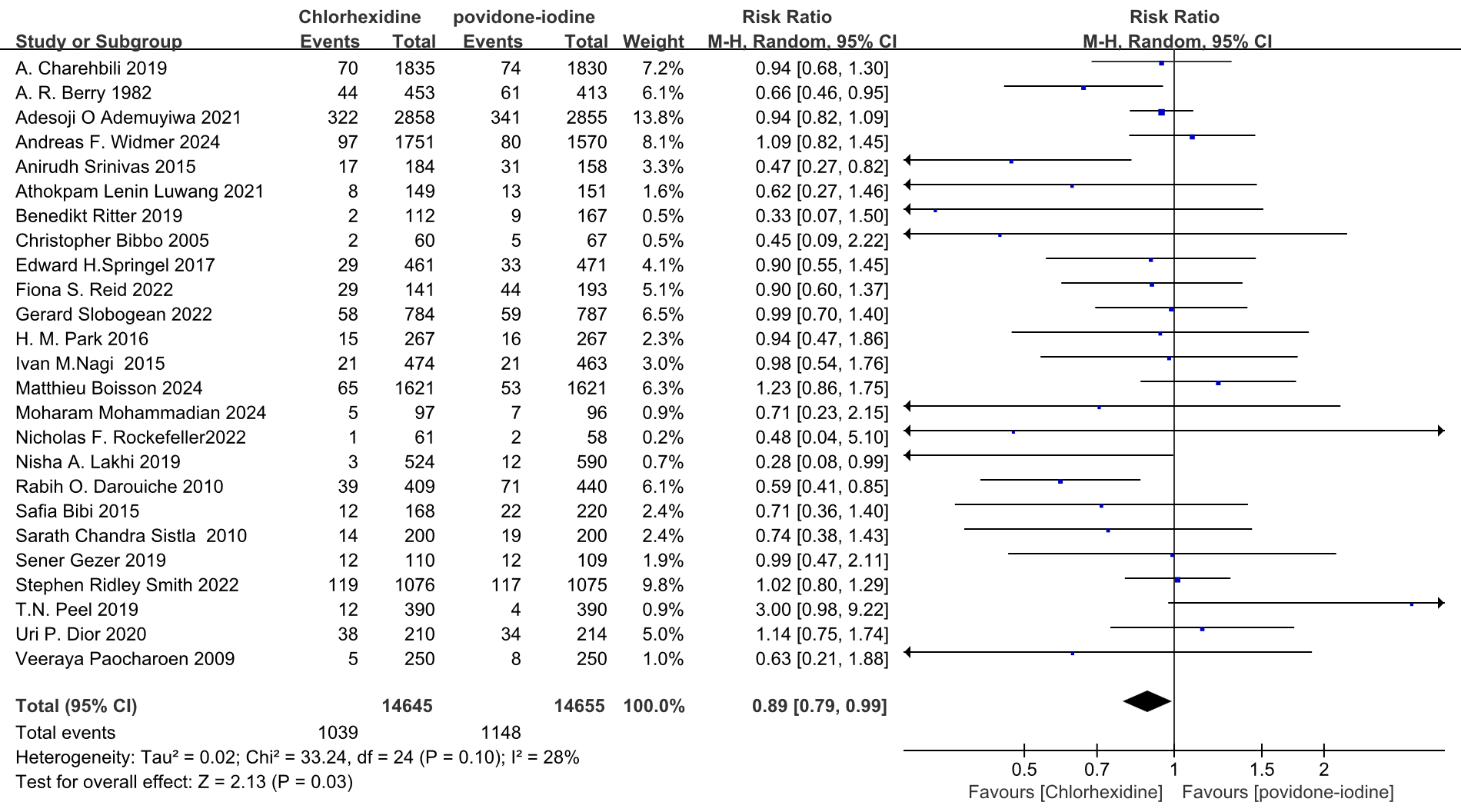


**Supplementary Figure 20**. Sensitivity analysis: Effect estimates after excluding one study with “some concerns”.
